# Measurements of top-quark pair differential cross-sections in the lepton+jets channel in *pp* collisions at $\sqrt{s}=8\,~{\mathrm {TeV}}$ using the ATLAS detector

**DOI:** 10.1140/epjc/s10052-016-4366-4

**Published:** 2016-10-03

**Authors:** G. Aad, B. Abbott, J. Abdallah, O. Abdinov, R. Aben, M. Abolins, O. S. AbouZeid, H. Abramowicz, H. Abreu, R. Abreu, Y. Abulaiti, B. S. Acharya, L. Adamczyk, D. L. Adams, J. Adelman, S. Adomeit, T. Adye, A. A. Affolder, T. Agatonovic-Jovin, J. Agricola, J. A. Aguilar-Saavedra, S. P. Ahlen, F. Ahmadov, G. Aielli, H. Akerstedt, T. P. A. Åkesson, A. V. Akimov, G. L. Alberghi, J. Albert, S. Albrand, M. J. Alconada Verzini, M. Aleksa, I. N. Aleksandrov, C. Alexa, G. Alexander, T. Alexopoulos, M. Alhroob, G. Alimonti, L. Alio, J. Alison, S. P. Alkire, B. M. M. Allbrooke, P. P. Allport, A. Aloisio, A. Alonso, F. Alonso, C. Alpigiani, A. Altheimer, B. Alvarez Gonzalez, D. Álvarez Piqueras, M. G. Alviggi, B. T. Amadio, K. Amako, Y. Amaral Coutinho, C. Amelung, D. Amidei, S. P. Amor Dos Santos, A. Amorim, S. Amoroso, N. Amram, G. Amundsen, C. Anastopoulos, L. S. Ancu, N. Andari, T. Andeen, C. F. Anders, G. Anders, J. K. Anders, K. J. Anderson, A. Andreazza, V. Andrei, S. Angelidakis, I. Angelozzi, P. Anger, A. Angerami, F. Anghinolfi, A. V. Anisenkov, N. Anjos, A. Annovi, M. Antonelli, A. Antonov, J. Antos, F. Anulli, M. Aoki, L. Aperio Bella, G. Arabidze, Y. Arai, J. P. Araque, A. T. H. Arce, F. A. Arduh, J-F. Arguin, S. Argyropoulos, M. Arik, A. J. Armbruster, O. Arnaez, H. Arnold, M. Arratia, O. Arslan, A. Artamonov, G. Artoni, S. Artz, S. Asai, N. Asbah, A. Ashkenazi, B. Åsman, L. Asquith, K. Assamagan, R. Astalos, M. Atkinson, N. B. Atlay, K. Augsten, M. Aurousseau, G. Avolio, B. Axen, M. K. Ayoub, G. Azuelos, M. A. Baak, A. E. Baas, M. J. Baca, C. Bacci, H. Bachacou, K. Bachas, M. Backes, M. Backhaus, P. Bagiacchi, P. Bagnaia, Y. Bai, T. Bain, J. T. Baines, O. K. Baker, E. M. Baldin, P. Balek, T. Balestri, F. Balli, W. K. Balunas, E. Banas, Sw. Banerjee, A. A. E. Bannoura, L. Barak, E. L. Barberio, D. Barberis, M. Barbero, T. Barillari, M. Barisonzi, T. Barklow, N. Barlow, S. L. Barnes, B. M. Barnett, R. M. Barnett, Z. Barnovska, A. Baroncelli, G. Barone, A. J. Barr, F. Barreiro, J. Barreiro Guimarães da Costa, R. Bartoldus, A. E. Barton, P. Bartos, A. Basalaev, A. Bassalat, A. Basye, R. L. Bates, S. J. Batista, J. R. Batley, M. Battaglia, M. Bauce, F. Bauer, H. S. Bawa, J. B. Beacham, M. D. Beattie, T. Beau, P. H. Beauchemin, R. Beccherle, P. Bechtle, H. P. Beck, K. Becker, M. Becker, M. Beckingham, C. Becot, A. J. Beddall, A. Beddall, V. A. Bednyakov, C. P. Bee, L. J. Beemster, T. A. Beermann, M. Begel, J. K. Behr, C. Belanger-Champagne, W. H. Bell, G. Bella, L. Bellagamba, A. Bellerive, M. Bellomo, K. Belotskiy, O. Beltramello, O. Benary, D. Benchekroun, M. Bender, K. Bendtz, N. Benekos, Y. Benhammou, E. Benhar Noccioli, J. A. Benitez Garcia, D. P. Benjamin, J. R. Bensinger, S. Bentvelsen, L. Beresford, M. Beretta, D. Berge, E. Bergeaas Kuutmann, N. Berger, F. Berghaus, J. Beringer, C. Bernard, N. R. Bernard, C. Bernius, F. U. Bernlochner, T. Berry, P. Berta, C. Bertella, G. Bertoli, F. Bertolucci, C. Bertsche, D. Bertsche, M. I. Besana, G. J. Besjes, O. Bessidskaia Bylund, M. Bessner, N. Besson, C. Betancourt, S. Bethke, A. J. Bevan, W. Bhimji, R. M. Bianchi, L. Bianchini, M. Bianco, O. Biebel, D. Biedermann, N. V. Biesuz, M. Biglietti, J. Bilbao De Mendizabal, H. Bilokon, M. Bindi, S. Binet, A. Bingul, C. Bini, S. Biondi, D. M. Bjergaard, C. W. Black, J. E. Black, K. M. Black, D. Blackburn, R. E. Blair, J.-B. Blanchard, J. E. Blanco, T. Blazek, I. Bloch, C. Blocker, W. Blum, U. Blumenschein, S. Blunier, G. J. Bobbink, V. S. Bobrovnikov, S. S. Bocchetta, A. Bocci, C. Bock, M. Boehler, J. A. Bogaerts, D. Bogavac, A. G. Bogdanchikov, C. Bohm, V. Boisvert, T. Bold, V. Boldea, A. S. Boldyrev, M. Bomben, M. Bona, M. Boonekamp, A. Borisov, G. Borissov, S. Borroni, J. Bortfeldt, V. Bortolotto, K. Bos, D. Boscherini, M. Bosman, J. Boudreau, J. Bouffard, E. V. Bouhova-Thacker, D. Boumediene, C. Bourdarios, N. Bousson, S. K. Boutle, A. Boveia, J. Boyd, I. R. Boyko, I. Bozic, J. Bracinik, A. Brandt, G. Brandt, O. Brandt, U. Bratzler, B. Brau, J. E. Brau, H. M. Braun, W. D. Breaden Madden, K. Brendlinger, A. J. Brennan, L. Brenner, R. Brenner, S. Bressler, T. M. Bristow, D. Britton, D. Britzger, F. M. Brochu, I. Brock, R. Brock, J. Bronner, G. Brooijmans, T. Brooks, W. K. Brooks, J. Brosamer, E. Brost, P. A. Bruckman de Renstrom, D. Bruncko, R. Bruneliere, A. Bruni, G. Bruni, M. Bruschi, N. Bruscino, L. Bryngemark, T. Buanes, Q. Buat, P. Buchholz, A. G. Buckley, I. A. Budagov, F. Buehrer, L. Bugge, M. K. Bugge, O. Bulekov, D. Bullock, H. Burckhart, S. Burdin, C. D. Burgard, B. Burghgrave, S. Burke, I. Burmeister, E. Busato, D. Büscher, V. Büscher, P. Bussey, J. M. Butler, A. I. Butt, C. M. Buttar, J. M. Butterworth, P. Butti, W. Buttinger, A. Buzatu, A. R. Buzykaev, S. Cabrera Urbán, D. Caforio, V. M. Cairo, O. Cakir, N. Calace, P. Calafiura, A. Calandri, G. Calderini, P. Calfayan, L. P. Caloba, D. Calvet, S. Calvet, R. Camacho Toro, S. Camarda, P. Camarri, D. Cameron, R. Caminal Armadans, S. Campana, M. Campanelli, A. Campoverde, V. Canale, A. Canepa, M. Cano Bret, J. Cantero, R. Cantrill, T. Cao, M. D. M. Capeans Garrido, I. Caprini, M. Caprini, M. Capua, R. Caputo, R. M. Carbone, R. Cardarelli, F. Cardillo, T. Carli, G. Carlino, L. Carminati, S. Caron, E. Carquin, G. D. Carrillo-Montoya, J. R. Carter, J. Carvalho, D. Casadei, M. P. Casado, M. Casolino, D. W. Casper, E. Castaneda-Miranda, A. Castelli, V. Castillo Gimenez, N. F. Castro, P. Catastini, A. Catinaccio, J. R. Catmore, A. Cattai, J. Caudron, V. Cavaliere, D. Cavalli, M. Cavalli-Sforza, V. Cavasinni, F. Ceradini, L. Cerda Alberich, B. C. Cerio, K. Cerny, A. S. Cerqueira, A. Cerri, L. Cerrito, F. Cerutti, M. Cerv, A. Cervelli, S. A. Cetin, A. Chafaq, D. Chakraborty, I. Chalupkova, Y. L. Chan, P. Chang, J. D. Chapman, D. G. Charlton, C. C. Chau, C. A. Chavez Barajas, S. Cheatham, A. Chegwidden, S. Chekanov, S. V. Chekulaev, G. A. Chelkov, M. A. Chelstowska, C. Chen, H. Chen, K. Chen, L. Chen, S. Chen, S. Chen, X. Chen, Y. Chen, H. C. Cheng, Y. Cheng, A. Cheplakov, E. Cheremushkina, R. Cherkaoui El Moursli, V. Chernyatin, E. Cheu, L. Chevalier, V. Chiarella, G. Chiarelli, G. Chiodini, A. S. Chisholm, R. T. Chislett, A. Chitan, M. V. Chizhov, K. Choi, S. Chouridou, B. K. B. Chow, V. Christodoulou, D. Chromek-Burckhart, J. Chudoba, A. J. Chuinard, J. J. Chwastowski, L. Chytka, G. Ciapetti, A. K. Ciftci, D. Cinca, V. Cindro, I. A. Cioara, A. Ciocio, F. Cirotto, Z. H. Citron, M. Ciubancan, A. Clark, B. L. Clark, P. J. Clark, R. N. Clarke, C. Clement, Y. Coadou, M. Cobal, A. Coccaro, J. Cochran, L. Coffey, J. G. Cogan, L. Colasurdo, B. Cole, S. Cole, A. P. Colijn, J. Collot, T. Colombo, G. Compostella, P. Conde Muiño, E. Coniavitis, S. H. Connell, I. A. Connelly, V. Consorti, S. Constantinescu, C. Conta, G. Conti, F. Conventi, M. Cooke, B. D. Cooper, A. M. Cooper-Sarkar, T. Cornelissen, M. Corradi, F. Corriveau, A. Corso-Radu, A. Cortes-Gonzalez, G. Cortiana, G. Costa, M. J. Costa, D. Costanzo, D. Côté, G. Cottin, G. Cowan, B. E. Cox, K. Cranmer, G. Cree, S. Crépé-Renaudin, F. Crescioli, W. A. Cribbs, M. Crispin Ortuzar, M. Cristinziani, V. Croft, G. Crosetti, T. Cuhadar Donszelmann, J. Cummings, M. Curatolo, J. Cúth, C. Cuthbert, H. Czirr, P. Czodrowski, S. D’Auria, M. D’Onofrio, M. J. Da Cunha Sargedas De Sousa, C. Da Via, W. Dabrowski, A. Dafinca, T. Dai, O. Dale, F. Dallaire, C. Dallapiccola, M. Dam, J. R. Dandoy, N. P. Dang, A. C. Daniells, M. Danninger, M. Dano Hoffmann, V. Dao, G. Darbo, S. Darmora, J. Dassoulas, A. Dattagupta, W. Davey, C. David, T. Davidek, E. Davies, M. Davies, P. Davison, Y. Davygora, E. Dawe, I. Dawson, R. K. Daya-Ishmukhametova, K. De, R. de Asmundis, A. De Benedetti, S. De Castro, S. De Cecco, N. De Groot, P. de Jong, H. De la Torre, F. De Lorenzi, D. De Pedis, A. De Salvo, U. De Sanctis, A. De Santo, J. B. De Vivie De Regie, W. J. Dearnaley, R. Debbe, C. Debenedetti, D. V. Dedovich, I. Deigaard, J. Del Peso, T. Del Prete, D. Delgove, F. Deliot, C. M. Delitzsch, M. Deliyergiyev, A. Dell’Acqua, L. Dell’Asta, M. Dell’Orso, M. Della Pietra, D. della Volpe, M. Delmastro, P. A. Delsart, C. Deluca, D. A. DeMarco, S. Demers, M. Demichev, A. Demilly, S. P. Denisov, D. Derendarz, J. E. Derkaoui, F. Derue, P. Dervan, K. Desch, C. Deterre, K. Dette, P. O. Deviveiros, A. Dewhurst, S. Dhaliwal, A. Di Ciaccio, L. Di Ciaccio, A. Di Domenico, C. Di Donato, A. Di Girolamo, B. Di Girolamo, A. Di Mattia, B. Di Micco, R. Di Nardo, A. Di Simone, R. Di Sipio, D. Di Valentino, C. Diaconu, M. Diamond, F. A. Dias, M. A. Diaz, E. B. Diehl, J. Dietrich, S. Diglio, A. Dimitrievska, J. Dingfelder, P. Dita, S. Dita, F. Dittus, F. Djama, T. Djobava, J. I. Djuvsland, M. A. B. do Vale, D. Dobos, M. Dobre, C. Doglioni, T. Dohmae, J. Dolejsi, Z. Dolezal, B. A. Dolgoshein, M. Donadelli, S. Donati, P. Dondero, J. Donini, J. Dopke, A. Doria, M. T. Dova, A. T. Doyle, E. Drechsler, M. Dris, Y. Du, E. Dubreuil, E. Duchovni, G. Duckeck, O. A. Ducu, D. Duda, A. Dudarev, L. Duflot, L. Duguid, M. Dührssen, M. Dunford, H. Duran Yildiz, M. Düren, A. Durglishvili, D. Duschinger, B. Dutta, M. Dyndal, C. Eckardt, K. M. Ecker, R. C. Edgar, W. Edson, N. C. Edwards, W. Ehrenfeld, T. Eifert, G. Eigen, K. Einsweiler, T. Ekelof, M. El Kacimi, M. Ellert, S. Elles, F. Ellinghaus, A. A. Elliot, N. Ellis, J. Elmsheuser, M. Elsing, D. Emeliyanov, Y. Enari, O. C. Endner, M. Endo, J. Erdmann, A. Ereditato, G. Ernis, J. Ernst, M. Ernst, S. Errede, E. Ertel, M. Escalier, H. Esch, C. Escobar, B. Esposito, A. I. Etienvre, E. Etzion, H. Evans, A. Ezhilov, L. Fabbri, G. Facini, R. M. Fakhrutdinov, S. Falciano, R. J. Falla, J. Faltova, Y. Fang, M. Fanti, A. Farbin, A. Farilla, T. Farooque, S. Farrell, S. M. Farrington, P. Farthouat, F. Fassi, P. Fassnacht, D. Fassouliotis, M. Faucci Giannelli, A. Favareto, L. Fayard, O. L. Fedin, W. Fedorko, S. Feigl, L. Feligioni, C. Feng, E. J. Feng, H. Feng, A. B. Fenyuk, L. Feremenga, P. Fernandez Martinez, S. Fernandez Perez, J. Ferrando, A. Ferrari, P. Ferrari, R. Ferrari, D. E. Ferreira de Lima, A. Ferrer, D. Ferrere, C. Ferretti, A. Ferretto Parodi, M. Fiascaris, F. Fiedler, A. Filipčič, M. Filipuzzi, F. Filthaut, M. Fincke-Keeler, K. D. Finelli, M. C. N. Fiolhais, L. Fiorini, A. Firan, A. Fischer, C. Fischer, J. Fischer, W. C. Fisher, N. Flaschel, I. Fleck, P. Fleischmann, G. T. Fletcher, G. Fletcher, R. R. M. Fletcher, T. Flick, A. Floderus, L. R. Flores Castillo, M. J. Flowerdew, A. Formica, A. Forti, D. Fournier, H. Fox, S. Fracchia, P. Francavilla, M. Franchini, D. Francis, L. Franconi, M. Franklin, M. Frate, M. Fraternali, D. Freeborn, S. T. French, S. M. Fressard-Batraneanu, F. Friedrich, D. Froidevaux, J. A. Frost, C. Fukunaga, E. Fullana Torregrosa, B. G. Fulsom, T. Fusayasu, J. Fuster, C. Gabaldon, O. Gabizon, A. Gabrielli, A. Gabrielli, G. P. Gach, S. Gadatsch, S. Gadomski, G. Gagliardi, P. Gagnon, C. Galea, B. Galhardo, E. J. Gallas, B. J. Gallop, P. Gallus, G. Galster, K. K. Gan, J. Gao, Y. Gao, Y. S. Gao, F. M. Garay Walls, F. Garberson, C. García, J. E. García Navarro, M. Garcia-Sciveres, R. W. Gardner, N. Garelli, V. Garonne, C. Gatti, A. Gaudiello, G. Gaudio, B. Gaur, L. Gauthier, P. Gauzzi, I. L. Gavrilenko, C. Gay, G. Gaycken, E. N. Gazis, P. Ge, Z. Gecse, C. N. P. Gee, Ch. Geich-Gimbel, M. P. Geisler, C. Gemme, M. H. Genest, C. Geng, S. Gentile, M. George, S. George, D. Gerbaudo, A. Gershon, S. Ghasemi, H. Ghazlane, B. Giacobbe, S. Giagu, V. Giangiobbe, P. Giannetti, B. Gibbard, S. M. Gibson, M. Gignac, M. Gilchriese, T. P. S. Gillam, D. Gillberg, G. Gilles, D. M. Gingrich, N. Giokaris, M. P. Giordani, F. M. Giorgi, F. M. Giorgi, P. F. Giraud, P. Giromini, D. Giugni, C. Giuliani, M. Giulini, B. K. Gjelsten, S. Gkaitatzis, I. Gkialas, E. L. Gkougkousis, L. K. Gladilin, C. Glasman, J. Glatzer, P. C. F. Glaysher, A. Glazov, M. Goblirsch-Kolb, J. R. Goddard, J. Godlewski, S. Goldfarb, T. Golling, D. Golubkov, A. Gomes, R. Gonçalo, J. Goncalves Pinto Firmino Da Costa, L. Gonella, S. González de la Hoz, G. Gonzalez Parra, S. Gonzalez-Sevilla, L. Goossens, P. A. Gorbounov, H. A. Gordon, I. Gorelov, B. Gorini, E. Gorini, A. Gorišek, E. Gornicki, A. T. Goshaw, C. Gössling, M. I. Gostkin, D. Goujdami, A. G. Goussiou, N. Govender, E. Gozani, H. M. X. Grabas, L. Graber, I. Grabowska-Bold, P. O. J. Gradin, P. Grafström, J. Gramling, E. Gramstad, S. Grancagnolo, V. Gratchev, H. M. Gray, E. Graziani, Z. D. Greenwood, C. Grefe, K. Gregersen, I. M. Gregor, P. Grenier, J. Griffiths, A. A. Grillo, K. Grimm, S. Grinstein, Ph. Gris, J.-F. Grivaz, S. Groh, J. P. Grohs, A. Grohsjean, E. Gross, J. Grosse-Knetter, G. C. Grossi, Z. J. Grout, L. Guan, J. Guenther, F. Guescini, D. Guest, O. Gueta, E. Guido, T. Guillemin, S. Guindon, U. Gul, C. Gumpert, J. Guo, Y. Guo, S. Gupta, G. Gustavino, P. Gutierrez, N. G. Gutierrez Ortiz, C. Gutschow, C. Guyot, C. Gwenlan, C. B. Gwilliam, A. Haas, C. Haber, H. K. Hadavand, N. Haddad, P. Haefner, S. Hageböck, Z. Hajduk, H. Hakobyan, M. Haleem, J. Haley, D. Hall, G. Halladjian, G. D. Hallewell, K. Hamacher, P. Hamal, K. Hamano, A. Hamilton, G. N. Hamity, P. G. Hamnett, L. Han, K. Hanagaki, K. Hanawa, M. Hance, B. Haney, P. Hanke, R. Hanna, J. B. Hansen, J. D. Hansen, M. C. Hansen, P. H. Hansen, K. Hara, A. S. Hard, T. Harenberg, F. Hariri, S. Harkusha, R. D. Harrington, P. F. Harrison, F. Hartjes, M. Hasegawa, Y. Hasegawa, A. Hasib, S. Hassani, S. Haug, R. Hauser, L. Hauswald, M. Havranek, C. M. Hawkes, R. J. Hawkings, A. D. Hawkins, T. Hayashi, D. Hayden, C. P. Hays, J. M. Hays, H. S. Hayward, S. J. Haywood, S. J. Head, T. Heck, V. Hedberg, L. Heelan, S. Heim, T. Heim, B. Heinemann, L. Heinrich, J. Hejbal, L. Helary, S. Hellman, C. Helsens, J. Henderson, R. C. W. Henderson, Y. Heng, C. Hengler, S. Henkelmann, A. Henrichs, A. M. Henriques Correia, S. Henrot-Versille, G. H. Herbert, Y. Hernández Jiménez, G. Herten, R. Hertenberger, L. Hervas, G. G. Hesketh, N. P. Hessey, J. W. Hetherly, R. Hickling, E. Higón-Rodriguez, E. Hill, J. C. Hill, K. H. Hiller, S. J. Hillier, I. Hinchliffe, E. Hines, R. R. Hinman, M. Hirose, D. Hirschbuehl, J. Hobbs, N. Hod, M. C. Hodgkinson, P. Hodgson, A. Hoecker, M. R. Hoeferkamp, F. Hoenig, M. Hohlfeld, D. Hohn, T. R. Holmes, M. Homann, T. M. Hong, W. H. Hopkins, Y. Horii, A. J. Horton, J-Y. Hostachy, S. Hou, A. Hoummada, J. Howard, J. Howarth, M. Hrabovsky, I. Hristova, J. Hrivnac, T. Hryn’ova, A. Hrynevich, C. Hsu, P. J. Hsu, S.-C. Hsu, D. Hu, Q. Hu, X. Hu, Y. Huang, Z. Hubacek, F. Hubaut, F. Huegging, T. B. Huffman, E. W. Hughes, G. Hughes, M. Huhtinen, T. A. Hülsing, N. Huseynov, J. Huston, J. Huth, G. Iacobucci, G. Iakovidis, I. Ibragimov, L. Iconomidou-Fayard, E. Ideal, Z. Idrissi, P. Iengo, O. Igonkina, T. Iizawa, Y. Ikegami, K. Ikematsu, M. Ikeno, Y. Ilchenko, D. Iliadis, N. Ilic, T. Ince, G. Introzzi, P. Ioannou, M. Iodice, K. Iordanidou, V. Ippolito, A. Irles Quiles, C. Isaksson, M. Ishino, M. Ishitsuka, R. Ishmukhametov, C. Issever, S. Istin, J. M. Iturbe Ponce, R. Iuppa, J. Ivarsson, W. Iwanski, H. Iwasaki, J. M. Izen, V. Izzo, S. Jabbar, B. Jackson, M. Jackson, P. Jackson, M. R. Jaekel, V. Jain, K. B. Jakobi, K. Jakobs, S. Jakobsen, T. Jakoubek, J. Jakubek, D. O. Jamin, D. K. Jana, E. Jansen, R. Jansky, J. Janssen, M. Janus, G. Jarlskog, N. Javadov, T. Javůrek, L. Jeanty, J. Jejelava, G.-Y. Jeng, D. Jennens, P. Jenni, J. Jentzsch, C. Jeske, S. Jézéquel, H. Ji, J. Jia, Y. Jiang, S. Jiggins, J. Jimenez Pena, S. Jin, A. Jinaru, O. Jinnouchi, M. D. Joergensen, P. Johansson, K. A. Johns, W. J. Johnson, K. Jon-And, G. Jones, R. W. L. Jones, T. J. Jones, J. Jongmanns, P. M. Jorge, K. D. Joshi, J. Jovicevic, X. Ju, A. Juste Rozas, M. Kaci, A. Kaczmarska, M. Kado, H. Kagan, M. Kagan, S. J. Kahn, E. Kajomovitz, C. W. Kalderon, A. Kaluza, S. Kama, A. Kamenshchikov, N. Kanaya, S. Kaneti, V. A. Kantserov, J. Kanzaki, B. Kaplan, L. S. Kaplan, A. Kapliy, D. Kar, K. Karakostas, A. Karamaoun, N. Karastathis, M. J. Kareem, E. Karentzos, M. Karnevskiy, S. N. Karpov, Z. M. Karpova, K. Karthik, V. Kartvelishvili, A. N. Karyukhin, K. Kasahara, L. Kashif, R. D. Kass, A. Kastanas, Y. Kataoka, C. Kato, A. Katre, J. Katzy, K. Kawade, K. Kawagoe, T. Kawamoto, G. Kawamura, S. Kazama, V. F. Kazanin, R. Keeler, R. Kehoe, J. S. Keller, J. J. Kempster, H. Keoshkerian, O. Kepka, B. P. Kerševan, S. Kersten, R. A. Keyes, F. Khalil-zada, H. Khandanyan, A. Khanov, A. G. Kharlamov, T. J. Khoo, V. Khovanskiy, E. Khramov, J. Khubua, S. Kido, H. Y. Kim, S. H. Kim, Y. K. Kim, N. Kimura, O. M. Kind, B. T. King, M. King, S. B. King, J. Kirk, A. E. Kiryunin, T. Kishimoto, D. Kisielewska, F. Kiss, K. Kiuchi, O. Kivernyk, E. Kladiva, M. H. Klein, M. Klein, U. Klein, K. Kleinknecht, P. Klimek, A. Klimentov, R. Klingenberg, J. A. Klinger, T. Klioutchnikova, E.-E. Kluge, P. Kluit, S. Kluth, J. Knapik, E. Kneringer, E. B. F. G. Knoops, A. Knue, A. Kobayashi, D. Kobayashi, T. Kobayashi, M. Kobel, M. Kocian, P. Kodys, T. Koffas, E. Koffeman, L. A. Kogan, S. Kohlmann, Z. Kohout, T. Kohriki, T. Koi, H. Kolanoski, M. Kolb, I. Koletsou, A. A. Komar, Y. Komori, T. Kondo, N. Kondrashova, K. Köneke, A. C. König, T. Kono, R. Konoplich, N. Konstantinidis, R. Kopeliansky, S. Koperny, L. Köpke, A. K. Kopp, K. Korcyl, K. Kordas, A. Korn, A. A. Korol, I. Korolkov, E. V. Korolkova, O. Kortner, S. Kortner, T. Kosek, V. V. Kostyukhin, V. M. Kotov, A. Kotwal, A. Kourkoumeli-Charalampidi, C. Kourkoumelis, V. Kouskoura, A. Koutsman, R. Kowalewski, T. Z. Kowalski, W. Kozanecki, A. S. Kozhin, V. A. Kramarenko, G. Kramberger, D. Krasnopevtsev, M. W. Krasny, A. Krasznahorkay, J. K. Kraus, A. Kravchenko, S. Kreiss, M. Kretz, J. Kretzschmar, K. Kreutzfeldt, P. Krieger, K. Krizka, K. Kroeninger, H. Kroha, J. Kroll, J. Kroseberg, J. Krstic, U. Kruchonak, H. Krüger, N. Krumnack, A. Kruse, M. C. Kruse, M. Kruskal, T. Kubota, H. Kucuk, S. Kuday, S. Kuehn, A. Kugel, F. Kuger, A. Kuhl, T. Kuhl, V. Kukhtin, R. Kukla, Y. Kulchitsky, S. Kuleshov, M. Kuna, T. Kunigo, A. Kupco, H. Kurashige, Y. A. Kurochkin, V. Kus, E. S. Kuwertz, M. Kuze, J. Kvita, T. Kwan, D. Kyriazopoulos, A. La Rosa, J. L. La Rosa Navarro, L. La Rotonda, C. Lacasta, F. Lacava, J. Lacey, H. Lacker, D. Lacour, V. R. Lacuesta, E. Ladygin, R. Lafaye, B. Laforge, T. Lagouri, S. Lai, L. Lambourne, S. Lammers, C. L. Lampen, W. Lampl, E. Lançon, U. Landgraf, M. P. J. Landon, V. S. Lang, J. C. Lange, A. J. Lankford, F. Lanni, K. Lantzsch, A. Lanza, S. Laplace, C. Lapoire, J. F. Laporte, T. Lari, F. Lasagni Manghi, M. Lassnig, P. Laurelli, W. Lavrijsen, A. T. Law, P. Laycock, T. Lazovich, O. Le Dortz, E. Le Guirriec, E. Le Menedeu, M. LeBlanc, T. LeCompte, F. Ledroit-Guillon, C. A. Lee, S. C. Lee, L. Lee, G. Lefebvre, M. Lefebvre, F. Legger, C. Leggett, A. Lehan, G. Lehmann Miotto, X. Lei, W. A. Leight, A. Leisos, A. G. Leister, M. A. L. Leite, R. Leitner, D. Lellouch, B. Lemmer, K. J. C. Leney, T. Lenz, B. Lenzi, R. Leone, S. Leone, C. Leonidopoulos, S. Leontsinis, C. Leroy, C. G. Lester, M. Levchenko, J. Levêque, D. Levin, L. J. Levinson, M. Levy, A. Lewis, A. M. Leyko, M. Leyton, B. Li, H. Li, H. L. Li, L. Li, L. Li, S. Li, X. Li, Y. Li, Z. Liang, H. Liao, B. Liberti, A. Liblong, P. Lichard, K. Lie, J. Liebal, W. Liebig, C. Limbach, A. Limosani, S. C. Lin, T. H. Lin, F. Linde, B. E. Lindquist, J. T. Linnemann, E. Lipeles, A. Lipniacka, M. Lisovyi, T. M. Liss, D. Lissauer, A. Lister, A. M. Litke, B. Liu, D. Liu, H. Liu, J. Liu, J. B. Liu, K. Liu, L. Liu, M. Liu, M. Liu, Y. Liu, M. Livan, A. Lleres, J. Llorente Merino, S. L. Lloyd, F. Lo Sterzo, E. Lobodzinska, P. Loch, W. S. Lockman, F. K. Loebinger, A. E. Loevschall-Jensen, K. M. Loew, A. Loginov, T. Lohse, K. Lohwasser, M. Lokajicek, B. A. Long, J. D. Long, R. E. Long, K. A. Looper, L. Lopes, D. Lopez Mateos, B. Lopez Paredes, I. Lopez Paz, J. Lorenz, N. Lorenzo Martinez, M. Losada, P. J. Lösel, X. Lou, A. Lounis, J. Love, P. A. Love, H. Lu, N. Lu, H. J. Lubatti, C. Luci, A. Lucotte, C. Luedtke, F. Luehring, W. Lukas, L. Luminari, O. Lundberg, B. Lund-Jensen, D. Lynn, R. Lysak, E. Lytken, H. Ma, L. L. Ma, G. Maccarrone, A. Macchiolo, C. M. Macdonald, B. Maček, J. Machado Miguens, D. Macina, D. Madaffari, R. Madar, H. J. Maddocks, W. F. Mader, A. Madsen, J. Maeda, S. Maeland, T. Maeno, A. Maevskiy, E. Magradze, K. Mahboubi, J. Mahlstedt, C. Maiani, C. Maidantchik, A. A. Maier, T. Maier, A. Maio, S. Majewski, Y. Makida, N. Makovec, B. Malaescu, Pa. Malecki, V. P. Maleev, F. Malek, U. Mallik, D. Malon, C. Malone, S. Maltezos, V. M. Malyshev, S. Malyukov, J. Mamuzic, G. Mancini, B. Mandelli, L. Mandelli, I. Mandić, R. Mandrysch, J. Maneira, L. Manhaes de Andrade Filho, J. Manjarres Ramos, A. Mann, A. Manousakis-Katsikakis, B. Mansoulie, R. Mantifel, M. Mantoani, L. Mapelli, L. March, G. Marchiori, M. Marcisovsky, C. P. Marino, M. Marjanovic, D. E. Marley, F. Marroquim, S. P. Marsden, Z. Marshall, L. F. Marti, S. Marti-Garcia, B. Martin, T. A. Martin, V. J. Martin, B. Martin dit Latour, M. Martinez, S. Martin-Haugh, V. S. Martoiu, A. C. Martyniuk, M. Marx, F. Marzano, A. Marzin, L. Masetti, T. Mashimo, R. Mashinistov, J. Masik, A. L. Maslennikov, I. Massa, L. Massa, P. Mastrandrea, A. Mastroberardino, T. Masubuchi, P. Mättig, J. Mattmann, J. Maurer, S. J. Maxfield, D. A. Maximov, R. Mazini, S. M. Mazza, G. Mc Goldrick, S. P. Mc Kee, A. McCarn, R. L. McCarthy, T. G. McCarthy, N. A. McCubbin, K. W. McFarlane, J. A. Mcfayden, G. Mchedlidze, S. J. McMahon, R. A. McPherson, M. Medinnis, S. Meehan, S. Mehlhase, A. Mehta, K. Meier, C. Meineck, B. Meirose, B. R. Mellado Garcia, F. Meloni, A. Mengarelli, S. Menke, E. Meoni, K. M. Mercurio, S. Mergelmeyer, P. Mermod, L. Merola, C. Meroni, F. S. Merritt, A. Messina, J. Metcalfe, A. S. Mete, C. Meyer, C. Meyer, J-P. Meyer, J. Meyer, H. Meyer Zu Theenhausen, R. P. Middleton, S. Miglioranzi, L. Mijović, G. Mikenberg, M. Mikestikova, M. Mikuž, M. Milesi, A. Milic, D. W. Miller, C. Mills, A. Milov, D. A. Milstead, A. A. Minaenko, Y. Minami, I. A. Minashvili, A. I. Mincer, B. Mindur, M. Mineev, Y. Ming, L. M. Mir, K. P. Mistry, T. Mitani, J. Mitrevski, V. A. Mitsou, A. Miucci, P. S. Miyagawa, J. U. Mjörnmark, T. Moa, K. Mochizuki, S. Mohapatra, W. Mohr, S. Molander, R. Moles-Valls, R. Monden, M. C. Mondragon, K. Mönig, C. Monini, J. Monk, E. Monnier, A. Montalbano, J. Montejo Berlingen, F. Monticelli, S. Monzani, R. W. Moore, N. Morange, D. Moreno, M. Moreno Llácer, P. Morettini, D. Mori, T. Mori, M. Morii, M. Morinaga, V. Morisbak, S. Moritz, A. K. Morley, G. Mornacchi, J. D. Morris, S. S. Mortensen, A. Morton, L. Morvaj, M. Mosidze, J. Moss, K. Motohashi, R. Mount, E. Mountricha, S. V. Mouraviev, E. J. W. Moyse, S. Muanza, R. D. Mudd, F. Mueller, J. Mueller, R. S. P. Mueller, T. Mueller, D. Muenstermann, P. Mullen, G. A. Mullier, F. J. Munoz Sanchez, J. A. Murillo Quijada, W. J. Murray, H. Musheghyan, E. Musto, A. G. Myagkov, M. Myska, B. P. Nachman, O. Nackenhorst, J. Nadal, K. Nagai, R. Nagai, Y. Nagai, K. Nagano, A. Nagarkar, Y. Nagasaka, K. Nagata, M. Nagel, E. Nagy, A. M. Nairz, Y. Nakahama, K. Nakamura, T. Nakamura, I. Nakano, H. Namasivayam, R. F. Naranjo Garcia, R. Narayan, D. I. Narrias Villar, T. Naumann, G. Navarro, R. Nayyar, H. A. Neal, P. Yu. Nechaeva, T. J. Neep, P. D. Nef, A. Negri, M. Negrini, S. Nektarijevic, C. Nellist, A. Nelson, S. Nemecek, P. Nemethy, A. A. Nepomuceno, M. Nessi, M. S. Neubauer, M. Neumann, R. M. Neves, P. Nevski, P. R. Newman, D. H. Nguyen, R. B. Nickerson, R. Nicolaidou, B. Nicquevert, J. Nielsen, N. Nikiforou, A. Nikiforov, V. Nikolaenko, I. Nikolic-Audit, K. Nikolopoulos, J. K. Nilsen, P. Nilsson, Y. Ninomiya, A. Nisati, R. Nisius, T. Nobe, M. Nomachi, I. Nomidis, T. Nooney, S. Norberg, M. Nordberg, O. Novgorodova, S. Nowak, M. Nozaki, L. Nozka, K. Ntekas, G. Nunes Hanninger, T. Nunnemann, E. Nurse, F. Nuti, F. O’grady, D. C. O’Neil, V. O’Shea, F. G. Oakham, H. Oberlack, T. Obermann, J. Ocariz, A. Ochi, I. Ochoa, J. P. Ochoa-Ricoux, S. Oda, S. Odaka, H. Ogren, A. Oh, S. H. Oh, C. C. Ohm, H. Ohman, H. Oide, W. Okamura, H. Okawa, Y. Okumura, T. Okuyama, A. Olariu, S. A. Olivares Pino, D. Oliveira Damazio, A. Olszewski, J. Olszowska, A. Onofre, K. Onogi, P. U. E. Onyisi, C. J. Oram, M. J. Oreglia, Y. Oren, D. Orestano, N. Orlando, C. Oropeza Barrera, R. S. Orr, B. Osculati, R. Ospanov, G. Otero y Garzon, H. Otono, M. Ouchrif, F. Ould-Saada, A. Ouraou, K. P. Oussoren, Q. Ouyang, A. Ovcharova, M. Owen, R. E. Owen, V. E. Ozcan, N. Ozturk, K. Pachal, A. Pacheco Pages, C. Padilla Aranda, M. Pagáčová, S. Pagan Griso, E. Paganis, F. Paige, P. Pais, K. Pajchel, G. Palacino, S. Palestini, M. Palka, D. Pallin, A. Palma, Y. B. Pan, E. St. Panagiotopoulou, C. E. Pandini, J. G. Panduro Vazquez, P. Pani, S. Panitkin, D. Pantea, L. Paolozzi, Th. D. Papadopoulou, K. Papageorgiou, A. Paramonov, D. Paredes Hernandez, M. A. Parker, K. A. Parker, F. Parodi, J. A. Parsons, U. Parzefall, E. Pasqualucci, S. Passaggio, F. Pastore, Fr. Pastore, G. Pásztor, S. Pataraia, N. D. Patel, J. R. Pater, T. Pauly, J. Pearce, B. Pearson, L. E. Pedersen, M. Pedersen, S. Pedraza Lopez, R. Pedro, S. V. Peleganchuk, D. Pelikan, O. Penc, C. Peng, H. Peng, B. Penning, J. Penwell, D. V. Perepelitsa, E. Perez Codina, M. T. Pérez García-Estañ, L. Perini, H. Pernegger, S. Perrella, R. Peschke, V. D. Peshekhonov, K. Peters, R. F. Y. Peters, B. A. Petersen, T. C. Petersen, E. Petit, A. Petridis, C. Petridou, P. Petroff, E. Petrolo, F. Petrucci, N. E. Pettersson, R. Pezoa, P. W. Phillips, G. Piacquadio, E. Pianori, A. Picazio, E. Piccaro, M. Piccinini, M. A. Pickering, R. Piegaia, D. T. Pignotti, J. E. Pilcher, A. D. Pilkington, A. W. J. Pin, J. Pina, M. Pinamonti, J. L. Pinfold, A. Pingel, S. Pires, H. Pirumov, M. Pitt, C. Pizio, L. Plazak, M.-A. Pleier, V. Pleskot, E. Plotnikova, P. Plucinski, D. Pluth, R. Poettgen, L. Poggioli, D. Pohl, G. Polesello, A. Poley, A. Policicchio, R. Polifka, A. Polini, C. S. Pollard, V. Polychronakos, K. Pommès, L. Pontecorvo, B. G. Pope, G. A. Popeneciu, D. S. Popovic, A. Poppleton, S. Pospisil, K. Potamianos, I. N. Potrap, C. J. Potter, C. T. Potter, G. Poulard, J. Poveda, V. Pozdnyakov, M. E. Pozo Astigarraga, P. Pralavorio, A. Pranko, S. Prasad, S. Prell, D. Price, L. E. Price, M. Primavera, S. Prince, M. Proissl, K. Prokofiev, F. Prokoshin, E. Protopapadaki, S. Protopopescu, J. Proudfoot, M. Przybycien, E. Ptacek, D. Puddu, E. Pueschel, D. Puldon, M. Purohit, P. Puzo, J. Qian, G. Qin, Y. Qin, A. Quadt, D. R. Quarrie, W. B. Quayle, M. Queitsch-Maitland, D. Quilty, S. Raddum, V. Radeka, V. Radescu, S. K. Radhakrishnan, P. Radloff, P. Rados, F. Ragusa, G. Rahal, S. Rajagopalan, M. Rammensee, C. Rangel-Smith, F. Rauscher, S. Rave, T. Ravenscroft, M. Raymond, A. L. Read, N. P. Readioff, D. M. Rebuzzi, A. Redelbach, G. Redlinger, R. Reece, K. Reeves, L. Rehnisch, J. Reichert, H. Reisin, C. Rembser, H. Ren, A. Renaud, M. Rescigno, S. Resconi, O. L. Rezanova, P. Reznicek, R. Rezvani, R. Richter, S. Richter, E. Richter-Was, O. Ricken, M. Ridel, P. Rieck, C. J. Riegel, J. Rieger, O. Rifki, M. Rijssenbeek, A. Rimoldi, L. Rinaldi, B. Ristić, E. Ritsch, I. Riu, F. Rizatdinova, E. Rizvi, S. H. Robertson, A. Robichaud-Veronneau, D. Robinson, J. E. M. Robinson, A. Robson, C. Roda, S. Roe, O. Røhne, A. Romaniouk, M. Romano, S. M. Romano Saez, E. Romero Adam, N. Rompotis, M. Ronzani, L. Roos, E. Ros, S. Rosati, K. Rosbach, P. Rose, O. Rosenthal, V. Rossetti, E. Rossi, L. P. Rossi, J. H. N. Rosten, R. Rosten, M. Rotaru, I. Roth, J. Rothberg, D. Rousseau, C. R. Royon, A. Rozanov, Y. Rozen, X. Ruan, F. Rubbo, I. Rubinskiy, V. I. Rud, C. Rudolph, M. S. Rudolph, F. Rühr, A. Ruiz-Martinez, Z. Rurikova, N. A. Rusakovich, A. Ruschke, H. L. Russell, J. P. Rutherfoord, N. Ruthmann, Y. F. Ryabov, M. Rybar, G. Rybkin, N. C. Ryder, A. Ryzhov, A. F. Saavedra, G. Sabato, S. Sacerdoti, A. Saddique, H. F-W. Sadrozinski, R. Sadykov, F. Safai Tehrani, P. Saha, M. Sahinsoy, M. Saimpert, T. Saito, H. Sakamoto, Y. Sakurai, G. Salamanna, A. Salamon, J. E. Salazar Loyola, M. Saleem, D. Salek, P. H. Sales De Bruin, D. Salihagic, A. Salnikov, J. Salt, D. Salvatore, F. Salvatore, A. Salvucci, A. Salzburger, D. Sammel, D. Sampsonidis, A. Sanchez, J. Sánchez, V. Sanchez Martinez, H. Sandaker, R. L. Sandbach, H. G. Sander, M. P. Sanders, M. Sandhoff, C. Sandoval, R. Sandstroem, D. P. C. Sankey, M. Sannino, A. Sansoni, C. Santoni, R. Santonico, H. Santos, I. Santoyo Castillo, K. Sapp, A. Sapronov, J. G. Saraiva, B. Sarrazin, O. Sasaki, Y. Sasaki, K. Sato, G. Sauvage, E. Sauvan, G. Savage, P. Savard, C. Sawyer, L. Sawyer, J. Saxon, C. Sbarra, A. Sbrizzi, T. Scanlon, D. A. Scannicchio, M. Scarcella, V. Scarfone, J. Schaarschmidt, P. Schacht, D. Schaefer, R. Schaefer, J. Schaeffer, S. Schaepe, S. Schaetzel, U. Schäfer, A. C. Schaffer, D. Schaile, R. D. Schamberger, V. Scharf, V. A. Schegelsky, D. Scheirich, M. Schernau, C. Schiavi, C. Schillo, M. Schioppa, S. Schlenker, K. Schmieden, C. Schmitt, S. Schmitt, S. Schmitt, S. Schmitz, B. Schneider, Y. J. Schnellbach, U. Schnoor, L. Schoeffel, A. Schoening, B. D. Schoenrock, E. Schopf, A. L. S. Schorlemmer, M. Schott, D. Schouten, J. Schovancova, S. Schramm, M. Schreyer, N. Schuh, M. J. Schultens, H.-C. Schultz-Coulon, H. Schulz, M. Schumacher, B. A. Schumm, Ph. Schune, C. Schwanenberger, A. Schwartzman, T. A. Schwarz, Ph. Schwegler, H. Schweiger, Ph. Schwemling, R. Schwienhorst, J. Schwindling, T. Schwindt, E. Scifo, G. Sciolla, F. Scuri, F. Scutti, J. Searcy, G. Sedov, E. Sedykh, P. Seema, S. C. Seidel, A. Seiden, F. Seifert, J. M. Seixas, G. Sekhniaidze, K. Sekhon, S. J. Sekula, D. M. Seliverstov, N. Semprini-Cesari, C. Serfon, L. Serin, L. Serkin, T. Serre, M. Sessa, R. Seuster, H. Severini, T. Sfiligoj, F. Sforza, A. Sfyrla, E. Shabalina, M. Shamim, L. Y. Shan, R. Shang, J. T. Shank, M. Shapiro, P. B. Shatalov, K. Shaw, S. M. Shaw, A. Shcherbakova, C. Y. Shehu, P. Sherwood, L. Shi, S. Shimizu, C. O. Shimmin, M. Shimojima, M. Shiyakova, A. Shmeleva, D. Shoaleh Saadi, M. J. Shochet, S. Shojaii, S. Shrestha, E. Shulga, M. A. Shupe, P. Sicho, P. E. Sidebo, O. Sidiropoulou, D. Sidorov, A. Sidoti, F. Siegert, Dj. Sijacki, J. Silva, Y. Silver, S. B. Silverstein, V. Simak, O. Simard, Lj. Simic, S. Simion, E. Simioni, B. Simmons, D. Simon, M. Simon, P. Sinervo, N. B. Sinev, M. Sioli, G. Siragusa, A. N. Sisakyan, S. Yu. Sivoklokov, J. Sjölin, T. B. Sjursen, M. B. Skinner, H. P. Skottowe, P. Skubic, M. Slater, T. Slavicek, M. Slawinska, K. Sliwa, V. Smakhtin, B. H. Smart, L. Smestad, S. Yu. Smirnov, Y. Smirnov, L. N. Smirnova, O. Smirnova, M. N. K. Smith, R. W. Smith, M. Smizanska, K. Smolek, A. A. Snesarev, G. Snidero, S. Snyder, R. Sobie, F. Socher, A. Soffer, D. A. Soh, G. Sokhrannyi, C. A. Solans, M. Solar, J. Solc, E. Yu. Soldatov, U. Soldevila, A. A. Solodkov, A. Soloshenko, O. V. Solovyanov, V. Solovyev, P. Sommer, H. Y. Song, N. Soni, A. Sood, A. Sopczak, B. Sopko, V. Sopko, V. Sorin, D. Sosa, M. Sosebee, C. L. Sotiropoulou, R. Soualah, A. M. Soukharev, D. South, B. C. Sowden, S. Spagnolo, M. Spalla, M. Spangenberg, F. Spanò, W. R. Spearman, D. Sperlich, F. Spettel, R. Spighi, G. Spigo, L. A. Spiller, M. Spousta, R. D. St. Denis, A. Stabile, S. Staerz, J. Stahlman, R. Stamen, S. Stamm, E. Stanecka, C. Stanescu, M. Stanescu-Bellu, M. M. Stanitzki, S. Stapnes, E. A. Starchenko, J. Stark, P. Staroba, P. Starovoitov, R. Staszewski, P. Steinberg, B. Stelzer, H. J. Stelzer, O. Stelzer-Chilton, H. Stenzel, G. A. Stewart, J. A. Stillings, M. C. Stockton, M. Stoebe, G. Stoicea, P. Stolte, S. Stonjek, A. R. Stradling, A. Straessner, M. E. Stramaglia, J. Strandberg, S. Strandberg, A. Strandlie, E. Strauss, M. Strauss, P. Strizenec, R. Ströhmer, D. M. Strom, R. Stroynowski, A. Strubig, S. A. Stucci, B. Stugu, N. A. Styles, D. Su, J. Su, R. Subramaniam, A. Succurro, S. Suchek, Y. Sugaya, M. Suk, V. V. Sulin, S. Sultansoy, T. Sumida, S. Sun, X. Sun, J. E. Sundermann, K. Suruliz, G. Susinno, M. R. Sutton, S. Suzuki, M. Svatos, M. Swiatlowski, I. Sykora, T. Sykora, D. Ta, C. Taccini, K. Tackmann, J. Taenzer, A. Taffard, R. Tafirout, N. Taiblum, H. Takai, R. Takashima, H. Takeda, T. Takeshita, Y. Takubo, M. Talby, A. A. Talyshev, J. Y. C. Tam, K. G. Tan, J. Tanaka, R. Tanaka, S. Tanaka, B. B. Tannenwald, S. Tapia Araya, S. Tapprogge, S. Tarem, F. Tarrade, G. F. Tartarelli, P. Tas, M. Tasevsky, T. Tashiro, E. Tassi, A. Tavares Delgado, Y. Tayalati, A. C. Taylor, F. E. Taylor, G. N. Taylor, P. T. E. Taylor, W. Taylor, F. A. Teischinger, M. Teixeira Dias Castanheira, P. Teixeira-Dias, K. K. Temming, D. Temple, H. Ten Kate, P. K. Teng, J. J. Teoh, F. Tepel, S. Terada, K. Terashi, J. Terron, S. Terzo, M. Testa, R. J. Teuscher, T. Theveneaux-Pelzer, J. P. Thomas, J. Thomas-Wilsker, E. N. Thompson, P. D. Thompson, R. J. Thompson, A. S. Thompson, L. A. Thomsen, E. Thomson, M. Thomson, R. P. Thun, M. J. Tibbetts, R. E. Ticse Torres, V. O. Tikhomirov, Yu. A. Tikhonov, S. Timoshenko, E. Tiouchichine, P. Tipton, S. Tisserant, K. Todome, T. Todorov, S. Todorova-Nova, J. Tojo, S. Tokár, K. Tokushuku, K. Tollefson, E. Tolley, L. Tomlinson, M. Tomoto, L. Tompkins, K. Toms, E. Torrence, H. Torres, E. Torró Pastor, J. Toth, F. Touchard, D. R. Tovey, T. Trefzger, L. Tremblet, A. Tricoli, I. M. Trigger, S. Trincaz-Duvoid, M. F. Tripiana, W. Trischuk, B. Trocmé, C. Troncon, M. Trottier-McDonald, M. Trovatelli, L. Truong, M. Trzebinski, A. Trzupek, C. Tsarouchas, J. C-L. Tseng, P. V. Tsiareshka, D. Tsionou, G. Tsipolitis, N. Tsirintanis, S. Tsiskaridze, V. Tsiskaridze, E. G. Tskhadadze, K. M. Tsui, I. I. Tsukerman, V. Tsulaia, S. Tsuno, D. Tsybychev, A. Tudorache, V. Tudorache, A. N. Tuna, S. A. Tupputi, S. Turchikhin, D. Turecek, R. Turra, A. J. Turvey, P. M. Tuts, A. Tykhonov, M. Tylmad, M. Tyndel, I. Ueda, R. Ueno, M. Ughetto, F. Ukegawa, G. Unal, A. Undrus, G. Unel, F. C. Ungaro, Y. Unno, C. Unverdorben, J. Urban, P. Urquijo, P. Urrejola, G. Usai, A. Usanova, L. Vacavant, V. Vacek, B. Vachon, C. Valderanis, N. Valencic, S. Valentinetti, A. Valero, L. Valery, S. Valkar, S. Vallecorsa, J. A. Valls Ferrer, W. Van Den Wollenberg, P. C. Van Der Deijl, R. van der Geer, H. van der Graaf, N. van Eldik, P. van Gemmeren, J. Van Nieuwkoop, I. van Vulpen, M. C. van Woerden, M. Vanadia, W. Vandelli, R. Vanguri, A. Vaniachine, F. Vannucci, G. Vardanyan, R. Vari, E. W. Varnes, T. Varol, D. Varouchas, A. Vartapetian, K. E. Varvell, F. Vazeille, T. Vazquez Schroeder, J. Veatch, L. M. Veloce, F. Veloso, T. Velz, S. Veneziano, A. Ventura, D. Ventura, M. Venturi, N. Venturi, A. Venturini, V. Vercesi, M. Verducci, W. Verkerke, J. C. Vermeulen, A. Vest, M. C. Vetterli, O. Viazlo, I. Vichou, T. Vickey, O. E. Vickey Boeriu, G. H. A. Viehhauser, S. Viel, R. Vigne, M. Villa, M. Villaplana Perez, E. Vilucchi, M. G. Vincter, V. B. Vinogradov, I. Vivarelli, S. Vlachos, D. Vladoiu, M. Vlasak, M. Vogel, P. Vokac, G. Volpi, M. Volpi, H. von der Schmitt, H. von Radziewski, E. von Toerne, V. Vorobel, K. Vorobev, M. Vos, R. Voss, J. H. Vossebeld, N. Vranjes, M. Vranjes Milosavljevic, V. Vrba, M. Vreeswijk, R. Vuillermet, I. Vukotic, Z. Vykydal, P. Wagner, W. Wagner, H. Wahlberg, S. Wahrmund, J. Wakabayashi, J. Walder, R. Walker, W. Walkowiak, C. Wang, F. Wang, H. Wang, H. Wang, J. Wang, J. Wang, K. Wang, R. Wang, S. M. Wang, T. Wang, T. Wang, X. Wang, C. Wanotayaroj, A. Warburton, C. P. Ward, D. R. Wardrope, A. Washbrook, C. Wasicki, P. M. Watkins, A. T. Watson, I. J. Watson, M. F. Watson, G. Watts, S. Watts, B. M. Waugh, S. Webb, M. S. Weber, S. W. Weber, J. S. Webster, A. R. Weidberg, B. Weinert, J. Weingarten, C. Weiser, H. Weits, P. S. Wells, T. Wenaus, T. Wengler, S. Wenig, N. Wermes, M. Werner, P. Werner, M. Wessels, J. Wetter, K. Whalen, A. M. Wharton, A. White, M. J. White, R. White, S. White, D. Whiteson, F. J. Wickens, W. Wiedenmann, M. Wielers, P. Wienemann, C. Wiglesworth, L. A. M. Wiik-Fuchs, A. Wildauer, H. G. Wilkens, H. H. Williams, S. Williams, C. Willis, S. Willocq, A. Wilson, J. A. Wilson, I. Wingerter-Seez, F. Winklmeier, B. T. Winter, M. Wittgen, J. Wittkowski, S. J. Wollstadt, M. W. Wolter, H. Wolters, B. K. Wosiek, J. Wotschack, M. J. Woudstra, K. W. Wozniak, M. Wu, M. Wu, S. L. Wu, X. Wu, Y. Wu, T. R. Wyatt, B. M. Wynne, S. Xella, D. Xu, L. Xu, B. Yabsley, S. Yacoob, R. Yakabe, M. Yamada, D. Yamaguchi, Y. Yamaguchi, A. Yamamoto, S. Yamamoto, T. Yamanaka, K. Yamauchi, Y. Yamazaki, Z. Yan, H. Yang, H. Yang, Y. Yang, W-M. Yao, Y. C. Yap, Y. Yasu, E. Yatsenko, K. H. Yau Wong, J. Ye, S. Ye, I. Yeletskikh, A. L. Yen, E. Yildirim, K. Yorita, R. Yoshida, K. Yoshihara, C. Young, C. J. S. Young, S. Youssef, D. R. Yu, J. Yu, J. M. Yu, J. Yu, L. Yuan, S. P. Y. Yuen, A. Yurkewicz, I. Yusuff, B. Zabinski, R. Zaidan, A. M. Zaitsev, J. Zalieckas, A. Zaman, S. Zambito, L. Zanello, D. Zanzi, C. Zeitnitz, M. Zeman, A. Zemla, J. C. Zeng, Q. Zeng, K. Zengel, O. Zenin, T. Ženiš, D. Zerwas, D. Zhang, F. Zhang, G. Zhang, H. Zhang, J. Zhang, L. Zhang, R. Zhang, X. Zhang, Z. Zhang, X. Zhao, Y. Zhao, Z. Zhao, A. Zhemchugov, J. Zhong, B. Zhou, C. Zhou, L. Zhou, L. Zhou, M. Zhou, N. Zhou, C. G. Zhu, H. Zhu, J. Zhu, Y. Zhu, X. Zhuang, K. Zhukov, A. Zibell, D. Zieminska, N. I. Zimine, C. Zimmermann, S. Zimmermann, Z. Zinonos, M. Zinser, M. Ziolkowski, L. Živković, G. Zobernig, A. Zoccoli, M. zur Nedden, G. Zurzolo, L. Zwalinski, ATLAS Collaboration

**Affiliations:** 1 Department of Physics, University of Adelaide, Adelaide, Australia; 2 Physics Department, SUNY Albany, Albany, NY USA; 3 Department of Physics, University of Alberta, Edmonton, AB Canada; 4 Department of Physics, Ankara University, Ankara, Turkey; 5 Istanbul Aydin University, Istanbul, Turkey; 6 Division of Physics, TOBB University of Economics and Technology, Ankara, Turkey; 7 LAPP, CNRS/IN2P3 and Université Savoie Mont Blanc, Annecy-le-Vieux, France; 8 High Energy Physics Division, Argonne National Laboratory, Argonne, IL USA; 9 Department of Physics, University of Arizona, Tucson, AZ USA; 10 Department of Physics, The University of Texas at Arlington, Arlington, TX USA; 11 Physics Department, University of Athens, Athens, Greece; 12 Physics Department, National Technical University of Athens, Zografou, Greece; 13 Institute of Physics, Azerbaijan Academy of Sciences, Baku, Azerbaijan; 14 Institut de Física d’Altes Energies and Departament de Física de la Universitat Autònoma de Barcelona, Barcelona, Spain; 15 Institute of Physics, University of Belgrade, Belgrade, Serbia; 16 Department for Physics and Technology, University of Bergen, Bergen, Norway; 17 Physics Division, Lawrence Berkeley National Laboratory and University of California, Berkeley, CA USA; 18 Department of Physics, Humboldt University, Berlin, Germany; 19 Albert Einstein Center for Fundamental Physics and Laboratory for High Energy Physics, University of Bern, Bern, Switzerland; 20 School of Physics and Astronomy, University of Birmingham, Birmingham, UK; 21 Department of Physics, Bogazici University, Istanbul, Turkey; 22 Department of Physics Engineering, Gaziantep University, Gaziantep, Turkey; 23 Department of Physics, Dogus University, Istanbul, Turkey; 24 INFN Sezione di Bologna, Bologna, Italy; 25 Dipartimento di Fisica e Astronomia, Università di Bologna, Bologna, Italy; 26 Physikalisches Institut, University of Bonn, Bonn, Germany; 27 Department of Physics, Boston University, Boston, MA USA; 28 Department of Physics, Brandeis University, Waltham, MA USA; 29 Universidade Federal do Rio De Janeiro COPPE/EE/IF, Rio de Janeiro, Brazil; 30 Electrical Circuits Department, Federal University of Juiz de Fora (UFJF), Juiz de Fora, Brazil; 31 Federal University of Sao Joao del Rei (UFSJ), Sao Joao del Rei, Brazil; 32 Instituto de Fisica, Universidade de Sao Paulo, Sao Paulo, Brazil; 33 Physics Department, Brookhaven National Laboratory, Upton, NY USA; 34 Transilvania University of Brasov, Brasov, Romania; 35 National Institute of Physics and Nuclear Engineering, Bucharest, Romania; 36 Physics Department, National Institute for Research and Development of Isotopic and Molecular Technologies, Cluj Napoca, Romania; 37 University Politehnica Bucharest, Bucharest, Romania; 38 West University in Timisoara, Timisoara, Romania; 39 Departamento de Física, Universidad de Buenos Aires, Buenos Aires, Argentina; 40 Cavendish Laboratory, University of Cambridge, Cambridge, UK; 41 Department of Physics, Carleton University, Ottawa, ON Canada; 42 CERN, Geneva, Switzerland; 43 Enrico Fermi Institute, University of Chicago, Chicago, IL USA; 44 Departamento de Física, Pontificia Universidad Católica de Chile, Santiago, Chile; 45 Departamento de Física, Universidad Técnica Federico Santa María, Valparaiso, Chile; 46 Institute of High Energy Physics, Chinese Academy of Sciences, Beijing, China; 47 Department of Modern Physics, University of Science and Technology of China, Hefei, Anhui China; 48 Department of Physics, Nanjing University, Jiangsu, China; 49 School of Physics, Shandong University, Shandong, China; 50 Department of Physics and Astronomy, Shanghai Key Laboratory for Particle Physics and Cosmology, Shanghai Jiao Tong University, Shanghai, China; 51 Physics Department, Tsinghua University, Beijing, 100084 China; 52 Laboratoire de Physique Corpusculaire, Clermont Université and Université Blaise Pascal and CNRS/IN2P3, Clermont-Ferrand, France; 53 Nevis Laboratory, Columbia University, Irvington, NY USA; 54 Niels Bohr Institute, University of Copenhagen, Copenhagen, Denmark; 55 INFN Gruppo Collegato di Cosenza, Laboratori Nazionali di Frascati, Rende, Italy; 56 Dipartimento di Fisica, Università della Calabria, Rende, Italy; 57 Faculty of Physics and Applied Computer Science, AGH University of Science and Technology, Kraków, Poland; 58 Marian Smoluchowski Institute of Physics, Jagiellonian University, Kraków, Poland; 59 Institute of Nuclear Physics, Polish Academy of Sciences, Kraków, Poland; 60 Physics Department, Southern Methodist University, Dallas, TX USA; 61 Physics Department, University of Texas at Dallas, Richardson, TX USA; 62 DESY, Hamburg and Zeuthen, Germany; 63 Institut für Experimentelle Physik IV, Technische Universität Dortmund, Dortmund, Germany; 64 Institut für Kern-und Teilchenphysik, Technische Universität Dresden, Dresden, Germany; 65 Department of Physics, Duke University, Durham, NC USA; 66 SUPA-School of Physics and Astronomy, University of Edinburgh, Edinburgh, UK; 67 INFN Laboratori Nazionali di Frascati, Frascati, Italy; 68 Fakultät für Mathematik und Physik, Albert-Ludwigs-Universität, Freiburg, Germany; 69 Section de Physique, Université de Genève, Geneva, Switzerland; 70 INFN Sezione di Genova, Genova, Italy; 71 Dipartimento di Fisica, Università di Genova, Genoa, Italy; 72 E. Andronikashvili Institute of Physics, Iv. Javakhishvili Tbilisi State University, Tbilisi, Georgia; 73 High Energy Physics Institute, Tbilisi State University, Tbilisi, Georgia; 74 II Physikalisches Institut, Justus-Liebig-Universität Giessen, Giessen, Germany; 75 SUPA-School of Physics and Astronomy, University of Glasgow, Glasgow, UK; 76 II Physikalisches Institut, Georg-August-Universität, Göttingen, Germany; 77 Laboratoire de Physique Subatomique et de Cosmologie, Université Grenoble-Alpes, CNRS/IN2P3, Grenoble, France; 78 Department of Physics, Hampton University, Hampton, VA USA; 79 Laboratory for Particle Physics and Cosmology, Harvard University, Cambridge, MA USA; 80 Kirchhoff-Institut für Physik, Ruprecht-Karls-Universität Heidelberg, Heidelberg, Germany; 81 Physikalisches Institut, Ruprecht-Karls-Universität Heidelberg, Heidelberg, Germany; 82 ZITI Institut für technische Informatik, Ruprecht-Karls-Universität Heidelberg, Mannheim, Germany; 83 Faculty of Applied Information Science, Hiroshima Institute of Technology, Hiroshima, Japan; 84 Department of Physics, The Chinese University of Hong Kong, Shatin, N.T. Hong Kong; 85 Department of Physics, The University of Hong Kong, Pokfulam, Hong Kong; 86 Department of Physics, The Hong Kong University of Science and Technology, Clear Water Bay, Kowloon, Hong Kong, China; 87 Department of Physics, Indiana University, Bloomington, IN USA; 88 Institut für Astro- und Teilchenphysik, Leopold-Franzens-Universität, Innsbruck, Austria; 89 University of Iowa, Iowa City, IA USA; 90 Department of Physics and Astronomy, Iowa State University, Ames, IA USA; 91 Joint Institute for Nuclear Research, JINR Dubna, Dubna, Russia; 92 KEK, High Energy Accelerator Research Organization, Tsukuba, Japan; 93 Graduate School of Science, Kobe University, Kobe, Japan; 94 Faculty of Science, Kyoto University, Kyoto, Japan; 95 Kyoto University of Education, Kyoto, Japan; 96 Department of Physics, Kyushu University, Fukuoka, Japan; 97 Instituto de Física La Plata, Universidad Nacional de La Plata and CONICET, La Plata, Argentina; 98 Physics Department, Lancaster University, Lancaster, UK; 99 INFN Sezione di Lecce, Lecce, Italy; 100 Dipartimento di Matematica e Fisica, Università del Salento, Lecce, Italy; 101 Oliver Lodge Laboratory, University of Liverpool, Liverpool, UK; 102 Department of Physics, Jožef Stefan Institute and University of Ljubljana, Ljubljana, Slovenia; 103 School of Physics and Astronomy, Queen Mary University of London, London, UK; 104 Department of Physics, Royal Holloway University of London, Surrey, UK; 105 Department of Physics and Astronomy, University College London, London, UK; 106 Louisiana Tech University, Ruston, LA USA; 107 Laboratoire de Physique Nucléaire et de Hautes Energies, UPMC and Université Paris-Diderot and CNRS/IN2P3, Paris, France; 108 Fysiska institutionen, Lunds universitet, Lund, Sweden; 109 Departamento de Fisica Teorica C-15, Universidad Autonoma de Madrid, Madrid, Spain; 110 Institut für Physik, Universität Mainz, Mainz, Germany; 111 School of Physics and Astronomy, University of Manchester, Manchester, UK; 112 CPP, MAix-Marseille Université and CNRS/IN2P3, Marseille, France; 113 Department of Physics, University of Massachusetts, Amherst, MA USA; 114 Department of Physics, McGill University, Montreal, QC Canada; 115 School of Physics, University of Melbourne, Melbourne, VIC Australia; 116 Department of Physics, The University of Michigan, Ann Arbor, MI USA; 117 Department of Physics and Astronomy, Michigan State University, East Lansing, MI USA; 118 INFN Sezione di Milano, Milan, Italy; 119 Dipartimento di Fisica, Università di Milano, Milan, Italy; 120 B.I. Stepanov Institute of Physics, National Academy of Sciences of Belarus, Minsk, Republic of Belarus; 121 National Scientific and Educational Centre for Particle and High Energy Physics, Minsk, Republic of Belarus; 122 Department of Physics, Massachusetts Institute of Technology, Cambridge, MA USA; 123 Group of Particle Physics, University of Montreal, Montreal, QC Canada; 124 P.N. Lebedev Physical Institute of the Russian, Academy of Sciences, Moscow, Russia; 125 Institute for Theoretical and Experimental Physics (ITEP), Moscow, Russia; 126 National Research Nuclear University MEPhI, Moscow, Russia; 127 D.V. Skobeltsyn Institute of Nuclear Physics, M.V. Lomonosov Moscow State University, Moscow, Russia; 128 Fakultät für Physik, Ludwig-Maximilians-Universität München, Munich, Germany; 129 Max-Planck-Institut für Physik (Werner-Heisenberg-Institut), Munich, Germany; 130 Nagasaki Institute of Applied Science, Nagasaki, Japan; 131 Graduate School of Science and Kobayashi-Maskawa Institute, Nagoya University, Nagoya, Japan; 132 INFN Sezione di Napoli, Napoles, Italy; 133 Dipartimento di Fisica, Università di Napoli, Naples, Italy; 134 Department of Physics and Astronomy, University of New Mexico, Albuquerque, NM USA; 135 Institute for MathematicsAstrophysics and Particle Physics, Radboud University Nijmegen/Nikhef, Nijmegen, The Netherlands; 136 Nikhef National Institute for Subatomic Physics and University of Amsterdam, Amsterdam, The Netherlands; 137 Department of Physics, Northern Illinois University, DeKalb, IL USA; 138 Budker Institute of Nuclear Physics, SB RAS, Novosibirsk, Russia; 139 Department of Physics, New York University, New York, NY USA; 140 Ohio State University, Columbus, OH USA; 141 Faculty of Science, Okayama University, Okayama, Japan; 142 Homer L. Dodge Department of Physics and Astronomy, University of Oklahoma, Norman, OK USA; 143 Department of Physics, Oklahoma State University, Stillwater, OK USA; 144 Palacký University, RCPTM, Olomouc, Czech Republic; 145 Center for High Energy Physics, University of Oregon, Eugene, OR USA; 146 LAL, Univ. Paris-Sud, CNRS/IN2P3, Université Paris-Saclay, Orsay, France; 147 Graduate School of Science, Osaka University, Osaka, Japan; 148 Department of Physics, University of Oslo, Oslo, Norway; 149 Department of Physics, Oxford University, Oxford, UK; 150 INFN Sezione di Pavia, Pavia, Italy; 151 Dipartimento di Fisica, Università di Pavia, Pavia, Italy; 152 Department of Physics, University of Pennsylvania, Philadelphia, PA USA; 153 National Research Centre “Kurchatov Institute” B.P. Konstantinov Petersburg Nuclear Physics Institute, St. Petersburg, Russia; 154 INFN Sezione di Pisa, Pisa, Italy; 155 Dipartimento di Fisica E. Fermi, Università di Pisa, Pisa, Italy; 156 Department of Physics and Astronomy, University of Pittsburgh, Pittsburgh, PA USA; 157 Laboratório de Instrumentação e Física Experimental de Partículas-LIP, Lisbon, Portugal; 158 Faculdade de Ciências, Universidade de Lisboa, Lisbon, Portugal; 159 Department of Physics, University of Coimbra, Coimbra, Portugal; 160 Centro de Física Nuclear da Universidade de Lisboa, Lisbon, Portugal; 161 Departamento de Fisica, Universidade do Minho, Braga, Portugal; 162 Departamento de Fisica Teorica y del Cosmos and CAFPE, Universidad de Granada, Granada, Spain; 163 Dep Fisica and CEFITEC of Faculdade de Ciencias e Tecnologia, Universidade Nova de Lisboa, Caparica, Portugal; 164 Institute of Physics, Academy of Sciences of the Czech Republic, Prague, Czech Republic; 165 Czech Technical University in Prague, Prague, Czech Republic; 166 Faculty of Mathematics and Physics, Charles University in Prague, Prague, Czech Republic; 167 State Research Center Institute for High Energy Physics (Protvino), NRC KI, Protvino, Russia; 168 Particle Physics Department, Rutherford Appleton Laboratory, Didcot, UK; 169 INFN Sezione di Roma, Rome, Italy; 170 Dipartimento di Fisica, Sapienza Università di Roma, Rome, Italy; 171 INFN Sezione di Roma Tor Vergata, Rome, Italy; 172 Dipartimento di Fisica, Università di Roma Tor Vergata, Rome, Italy; 173 INFN Sezione di Roma Tre, Rome, Italy; 174 Dipartimento di Matematica e Fisica, Università Roma Tre, Rome, Italy; 175 Faculté des Sciences Ain Chock, Réseau Universitaire de Physique des Hautes Energies-Université Hassan II, Casablanca, Morocco; 176 Centre National de l’Energie des Sciences Techniques Nucleaires, Rabat, Morocco; 177 Faculté des Sciences Semlalia, Université Cadi Ayyad, LPHEA-Marrakech, Marrakesh, Morocco; 178 Faculté des Sciences, Université Mohamed Premier and LPTPM, Oujda, Morocco; 179 Faculté des , Sciences, Université Mohammed V, Rabat, Morocco; 180 DSM/IRFU (Institut de Recherches sur les Lois Fondamentales de l’Univers), CEA Saclay (Commissariat à l’Energie Atomique et aux Energies Alternatives), Gif-sur-Yvette, France; 181 Santa Cruz Institute for Particle Physics, University of California Santa Cruz, Santa Cruz, CA USA; 182 Department of Physics, University of Washington, Seattle, WA USA; 183 Department of Physics and Astronomy, University of Sheffield, Sheffield, UK; 184 Department of Physics, Shinshu University, Nagano, Japan; 185 Fachbereich Physik, Universität Siegen, Siegen, Germany; 186 Department of Physics, Simon Fraser University, Burnaby, BC Canada; 187 SLAC National Accelerator Laboratory, Stanford, CA USA; 188 Faculty of Mathematics, Physics and Informatics, Comenius University, Bratislava, Slovakia; 189 Department of Subnuclear Physics, Institute of Experimental Physics of the Slovak Academy of Sciences, Kosice, Slovak Republic; 190 Department of Physics, University of Cape Town, Cape Town, South Africa; 191 Department of Physics, University of Johannesburg, Johannesburg, South Africa; 192 School of Physics, University of the Witwatersrand, Johannesburg, South Africa; 193 Department of Physics, Stockholm University, Stockholm, Sweden; 194 The Oskar Klein Centre, Stockholm, Sweden; 195 Physics Department, Royal Institute of Technology, Stockholm, Sweden; 196 Departments of Physics and Astronomy and Chemistry, Stony Brook University, Stony Brook, NY USA; 197 Department of Physics and Astronomy, University of Sussex, Brighton, UK; 198 School of Physics, University of Sydney, Sydney, Australia; 199 Institute of Physics, Academia Sinica, Taipei, Taiwan; 200 Department of Physics, Technion: Israel Institute of Technology, Haifa, Israel; 201 Raymond and Beverly Sackler School of Physics and Astronomy, Tel Aviv University, Tel Aviv, Israel; 202 Department of Physics, Aristotle University of Thessaloniki, Thessaloníki, Greece; 203 International Center for Elementary Particle Physics and Department of Physics, The University of Tokyo, Tokyo, Japan; 204 Graduate School of Science and Technology, Tokyo Metropolitan University, Tokyo, Japan; 205 Department of Physics, Tokyo Institute of Technology, Tokyo, Japan; 206 Department of Physics, University of Toronto, Toronto, ON Canada; 207 TRIUMF, Vancouver, BC Canada; 208 Department of Physics and Astronomy, York University, Toronto, ON Canada; 209 Faculty of Pure and Applied Sciences, and Center for Integrated Research in Fundamental Science and Engineering, University of Tsukuba, Tsukuba, Japan; 210 Department of Physics and Astronomy, Tufts University, Medford, MA USA; 211 Centro de Investigaciones, Universidad Antonio Narino, Bogota, Colombia; 212 Department of Physics and Astronomy, University of California Irvine, Irvine, CA USA; 213 INFN Gruppo Collegato di Udine, Sezione di Trieste, Udine, Italy; 214 ICTP, Trieste, Italy; 215 Dipartimento di Chimica, Fisica e Ambiente, Università di Udine, Udine, Italy; 216 Department of Physics, University of Illinois, Urbana, IL USA; 217 Department of Physics and Astronomy, University of Uppsala, Uppsala, Sweden; 218 Instituto de Física Corpuscular (IFIC) and Departamento de Física Atómica, Molecular y Nuclear and Departamento de Ingeniería Electrónica and Instituto de Microelectrónica de Barcelona (IMB-CNM), University of Valencia and CSIC, Valencia, Spain; 219 Department of Physics, University of British Columbia, Vancouver, BC Canada; 220 Department of Physics and Astronomy, University of Victoria, Victoria, BC Canada; 221 Department of Physics, University of Warwick, Coventry, UK; 222 Waseda University, Tokyo, Japan; 223 Department of Particle Physics, The Weizmann Institute of Science, Rehovot, Israel; 224 Department of Physics, University of Wisconsin, Madison, WI USA; 225 Fakultät für Physik und Astronomie, Julius-Maximilians-Universität, Würzburg, Germany; 226 Fachbereich C Physik, Bergische Universität Wuppertal, Wuppertal, Germany; 227 Department of Physics, Yale University, New Haven, CT USA; 228 Yerevan Physics Institute, Yerevan, Armenia; 229 Centre de Calcul de l’Institut National de Physique Nucléaire et de Physique des Particules (IN2P3), Villeurbanne, France

## Abstract

Measurements of normalized differential cross-sections of top-quark pair production are presented as a function of the top-quark, $t\bar{t}$ system and event-level kinematic observables in proton–proton collisions at a centre-of-mass energy of $\sqrt{s}=8\,\mathrm{TeV}$. The observables have been chosen to emphasize the $t\bar{t}$ production process and to be sensitive to effects of initial- and final-state radiation, to the different parton distribution functions, and to non-resonant processes and higher-order corrections. The dataset corresponds to an integrated luminosity of 20.3 fb$^{-1}$, recorded in 2012 with the ATLAS detector at the CERN Large Hadron Collider. Events are selected in the lepton+jets channel, requiring exactly one charged lepton and at least four jets with at least two of the jets tagged as originating from a *b*-quark. The measured spectra are corrected for detector effects and are compared to several Monte Carlo simulations. The results are in fair agreement with the predictions over a wide kinematic range. Nevertheless, most generators predict a harder top-quark transverse momentum distribution at high values than what is observed in the data. Predictions beyond NLO accuracy improve the agreement with data at high top-quark transverse momenta. Using the current settings and parton distribution functions, the rapidity distributions are not well modelled by any generator under consideration. However, the level of agreement is improved when more recent sets of parton distribution functions are used.

## Introduction

The large top-quark pair production cross-section at the LHC allows detailed studies of the characteristics of $t\bar{t}{}$ production to be performed with respect to different kinematic variables, providing a unique opportunity to test the Standard Model (SM) at the  ${\mathrm {TeV}}$ scale. Furthermore, effects beyond the SM can appear as modifications of $t\bar{t}{}$ differential distributions with respect to the SM predictions [1] which may not be detectable with an inclusive cross-section measurement. A precise measurement of the $t\bar{t}{}$ differential cross-section therefore has the potential to enhance the sensitivity to possible effects beyond the SM, as well as to clarify the ability of the theoretical calculations in describing the cross-section.

The ATLAS [2–4] and CMS [5] experiments have published measurements of the $t\bar{t}{}$ differential cross-sections at a centre-of-mass energy $\sqrt{s}=7$  ${\mathrm {TeV}}$ in *pp* collisions, both in the full phase space using parton-level variables and in fiducial phase-space regions using observables constructed from final-state particles (particle level); the CMS experiment also published measurements of the $t\bar{t}{}$ differential cross-sections with data taken at $\sqrt{s}=8$  ${\mathrm {TeV}}$ [6]. The results presented here represent the natural extension of the previous ATLAS measurements of the $t\bar{t}{}$ differential cross-sections to the $\sqrt{s}=8$  ${\mathrm {TeV}}$ dataset, and benefit from higher statistics and reduced detector uncertainties.

In the SM, the top quark decays almost exclusively into a $W$ boson and a *b*-quark. The signature of a $t\bar{t}$ decay is therefore determined by the $W$ boson decay modes. This analysis makes use of the lepton $+$ jets $t\bar{t}$ decay mode, where one $W$ boson decays into an electron or a muon and a neutrino and the other $W$ boson decays into a pair of quarks, with the two decay modes referred to as the *e*+jets and $\mu$+jets channel, respectively. Events in which the $W$ boson decays to an electron or muon through a $\tau$ lepton decay are also included.

This paper presents a set of measurements of the $t\bar{t}$ production cross-section as a function of different properties of the reconstructed top quark and of the $t\bar{t}$ system. The results, unfolded both to a fiducial particle-level phase space and to the full phase space, are compared to the predictions of Monte Carlo (MC) generators and to perturbative QCD calculations beyond the next-to-leading-order (NLO) approximation. The goal of unfolding to a fiducial particle-level phase space and of using variables directly related to detector observables is to allow precision tests of QCD, avoiding large model-dependent extrapolation corrections to the parton-level top-quark and to a phase space region outside the detector sensitivity. However, full phase-space measurements represent a valid test of higher-order calculations for which event generation with subsequent parton showering and hadronization is not yet available. A subset of the observables under consideration has been measured by CMS [5].

In addition to the variables measured at $\sqrt{s}=$7  ${\mathrm {TeV}}$ [2–4], a set of new measurements is presented. These variables, similar to those used in dijet measurements at large jet transverse momentum [7, 8], are sensitive to effects of initial- and final-state radiation, to the different parton distribution functions (PDF), and to non-resonant processes including particles beyond the Standard Model [9]. Finally, observables constructed as a function of the transverse momenta of the *W* boson and the *b*-quark originating from the top quark have been found to be sensitive to non-resonant effects (when one or both top-quarks are off-shell) [10] and non-factorizable higher-order corrections [11].

The paper is organized as follows: Sect. 2 briefly describes the ATLAS detector, while Sect. 3 describes the data and simulation samples used in the measurements. The reconstruction of physics objects and the event selection is explained in Sect. 4. Section  5 describes the kinematic reconstruction of the ${t\bar{t}}$ pairs using the pseudo-top algorithm. Section 6 discusses the background processes affecting these measurements. Event yields for both the signal and background samples, as well as distributions of measured quantities before unfolding, are shown in Sect. 7. The measurements of the cross-sections are described in Sect. 8. Statistical and systematic uncertainties are discussed in Sect. 9. The results are presented in Sect. 10, where the comparison with theoretical predictions is also discussed. Finally, a summary is presented in Sect. 11.

## The ATLAS detector

ATLAS is a multi-purpose detector [12] that provides nearly full solid angle coverage around the interaction point. This analysis exploits all major components of the detector. Charged-particle trajectories with pseudorapidity^1^ $|\eta | <2.5$ are reconstructed in the inner detector, which comprises a silicon pixel detector, a silicon microstrip detector and a transition radiation tracker (TRT). The inner detector is embedded in a 2 T axial magnetic field. Sampling calorimeters with several different designs span the pseudorapidity range up to $|\eta | = 4.9$. High-granularity liquid argon (LAr) electromagnetic (EM) calorimeters are available up to $|\eta | = 3.2$. Hadronic calorimeters based on scintillator-tile active material cover $|\eta | < 1.7$ while LAr technology is used for hadronic calorimetry from $|\eta | = 1.5$ to $|\eta | = 4.9$. The calorimeters are surrounded by a muon spectrometer within a magnetic field provided by air-core toroid magnets with a bending integral of about 2.5 Tm in the barrel and up to 6 Tm in the endcaps. Three stations of precision drift tubes and cathode-strip chambers provide an accurate measurement of the muon track curvature in the region $|\eta | < 2.7$. Resistive-plate and thin-gap chambers provide muon triggering capability up to $|\eta | = 2.4$.

Data are selected from inclusive *pp* interactions using a three-level trigger system. A hardware-based trigger (L1) uses custom-made hardware and low-granularity detector data to initially reduce the trigger rate to approximately 75 kHz. The detector readout is then available for two stages of software-based triggers. In the second level (L2), the trigger has access to the full detector granularity, but only retrieves data for regions of the detector identified by L1 as containing interesting objects. Finally, the Event Filter (EF) system makes use of the full detector readout to finalize the event selection. During the 2012 run period, the selected event rate for all triggers following the event filter was approximately 400 Hz.

## Data and simulation samples

The differential cross-sections are measured using a dataset collected by the ATLAS detector during the 2012 LHC *pp* run at $\sqrt{s}=8$ TeV, which corresponds to an integrated luminosity of $20.3~\pm ~0.6$ fb${^{-1}}$. The luminosity is measured using techniques similar to those described in Ref. [13] with a calibration of the luminosity scale derived from beam-separation scans. The average number of interactions per bunch crossing in 2012 was 21. Data events are considered only if they are acquired under stable beam conditions and with all sub-detectors operational. The data sample is collected using single-lepton triggers; for each lepton type the logical OR of two triggers is used in order to increase the efficiency for isolated leptons at low transverse momentum. The triggers with the lower $p_{\mathrm {T}}$ thresholds include isolation requirements on the candidate lepton, resulting in inefficiencies at high $p_{\mathrm {T}}$ that are recovered by the triggers with higher $p_{\mathrm {T}}$ thresholds. For electrons the two transverse momentum thresholds are 24 and 60 ${\mathrm {GeV}}$ while for muons the thresholds are 24 and 36 ${\mathrm {GeV}}$.

Simulated samples are used to characterize the detector response and efficiency to reconstruct $t\bar{t}$ events, estimate systematic uncertainties and predict the background contributions from various processes. The response of the detector is simulated [14] using a detailed model implemented in GEANT4 [15]. For the evaluation of some systematic uncertainties, generated samples are passed through a fast simulation using a parameterization of the performance of the ATLAS electromagnetic and hadronic calorimeters [16]. Simulated events include the effect of multiple *pp* collisions from the same and previous bunch-crossings (in-time and out-of-time pile-up) and are re-weighted to match the same number of collisions as observed in data. All simulated samples are normalized to the integrated luminosity of the data sample; in the normalization procedure the most precise cross-section calculations available are used.

The nominal signal $t\bar{t}$ sample is generated using the Powheg-Box  [17] generator, based on next-to-leading-order QCD matrix elements. The CT10 [18] parton distribution functions are employed and the top-quark mass ($m_{t}$) is set to 172.5 ${\mathrm {GeV}}$. The $h_\mathrm{damp}$  parameter, which effectively regulates the high-$p_{\mathrm {T}}$ radiation in Powheg, is set to the top-quark mass. Parton showering and hadronization are simulated with Pythia  [19] (version 6.427) using the Perugia 2011C set of tuned parameters [20]. The effect of the systematic uncertainties related to the PDF for the signal simulation are evaluated using samples generated with MC@NLO [21] (version 4.01) using the CT10nlo PDF set, interfaced to Herwig [22] (version 6.520) for parton showering and hadronization, and Jimmy [23] (version 4.31) for the modelling of multiple parton scattering. For the evaluation of systematic uncertainties due to the parton showering model, a Powheg +Herwig  sample is compared to a Powheg +Pythia  sample. The $h_\mathrm{damp}$  parameter in the Powheg +Herwig  sample is set to infinity. The uncertainties due to QCD initial- and final-state radiation (ISR/FSR) modelling are estimated with samples generated with Powheg-Box interfaced to Pythia for which the parameters of the generation ($\Lambda _\mathrm{QCD}$, $Q^2_\mathrm{max}$ scale, transverse momentum scale for space-like parton-shower evolution and the $h_\mathrm{damp}$  parameter) are varied to span the ranges compatible with the results of measurements of $t\bar{t}$ production in association with jets [24–26]. Finally, two additional $t\bar{t}$ samples are used only in the comparison against data. The first one is a sample of Powheg matrix elements generated with the nominal settings interfaced to Pythia8  [27] (version 8.186 and Main31 user hook) and the AU14 [28] set of tuned parameters. In the second sample, MadGraph  [29] ${t\bar{t}}$ matrix elements with up to three additional partons are interfaced to Pythia using the matrix-element to parton-shower MLM matching scheme [30] and the Perugia 2011C set of tuned parameters [20].

The $t\bar{t}$ samples are normalized to the NNLO+NNLL cross-section of $\sigma _{t\bar{t}}=253^{+13}_{-15}$ pb (scale, PDF and $\alpha _S$), evaluated using the Top++2.0 program [31], which includes the next-to-next-to-leading-order QCD corrections and resums next-to-next-to-leading logarithmic soft gluon terms [32–37]. The quoted cross-section corresponds to a top-quark mass of 172.5 ${\mathrm {GeV}}$. Each $t\bar{t}$ sample is produced requiring at least one semileptonic decay in the ${t\bar{t}}$ pair.

Single-top-quark processes for the *s*-channel, *t*-channel and *Wt* associated production constitute the largest background in this analysis. These processes are simulated with Powheg-Box using the PDF set CT10 and showered with Pythia (version 6.427) calibrated with the P2011C tune [20] and the PDF set CTEQ6L1 [38]. All possible production channels containing one lepton in the final state are considered. All samples are generated requiring the presence of a leptonically decaying *W* boson. The cross-sections multiplied by the branching ratios for the leptonic *W* decay employed for these processes are normalized to NLO+NNLL calculations [39–41].

Leptonic decays of vector bosons produced in association with high-$p_{\mathrm {T}}$ jets, referred to as *W*+jets and *Z*+jets, constitute the second largest background in this analysis. Samples of simulated *W* / *Z*+jets events with up to five additional partons in the LO matrix elements are produced with the Alpgen generator (version 2.13) [42] using the PDF set CTEQ6L1 [38] and interfaced to Pythia (version 6.427) for parton showering; the overlap between samples is dealt with by using the MLM matching scheme [30]. Heavy-flavour quarks are included in the matrix-element calculations to produce the $Wb\bar{b}$, $Wc\bar{c}$, *Wc*, $Zb\bar{b}$ and $Zc\bar{c}$ samples. The overlap between the heavy-flavour quarks produced by the matrix element and by the parton shower is removed. The *W*+jets samples are normalized to the inclusive *W* boson NNLO cross-section [43, 44] and corrected by applying additional scale factors derived from data, as described in Sect. 6.

Diboson production is modelled using Herwig and Jimmy with the CTEQ6L1 PDF set [38] and the yields are normalized using the NLO cross-sections [45]. All possible production channels containing at least one lepton in the final states are considered.

## Object definition and event selection

The lepton+jets $t\bar{t}$ decay mode is characterized by the presence of a high-$p_{\mathrm {T}}$ lepton, missing transverse momentum due to the neutrino, two jets originating from *b*-quarks, and two jets from the hadronic $W$ boson decay.

The following sections describe the detector-level, particle-level and parton-level objects used to characterize the final-state event topology and to define a fiducial phase-space region for the measurements.

### Detector-level objects

Primary vertices in the event are formed from reconstructed tracks such that they are spatially compatible with the luminous interaction region. The hard-scatter primary vertex is chosen to be the vertex with the highest $\sum p_{\mathrm {T}}^2$ where the sum extends over all associated tracks with $p_{\mathrm {T}}> 0.4\,\mathrm {{\mathrm {GeV}}{}}$.

Electron candidates are reconstructed by associating tracks in the inner detector with energy deposits in the EM calorimeter. They must satisfy identification criteria based on the shower shape in the EM calorimeter, on the track quality, and on the detection of the transition radiation produced in the TRT detector. The EM clusters are required to be in the pseudorapidity region $|\eta | < 2.47$, excluding the transition region between the barrel and the endcap calorimeters ($1.37< |\eta | < 1.52$). They must have a transverse energy $E_{\mathrm {T}}>25 \,$ ${\mathrm {GeV}}$. The associated track must have a longitudinal impact parameter $|z_0|<2$ mm with respect to the primary vertex. Isolation requirements, on calorimeter and tracking variables, are used to reduce the background from non-prompt electrons. The calorimeter isolation variable is based on the energy sum of cells within a cone of size $\Delta R < 0.2$ around the direction of each electron candidate. This energy sum excludes cells associated with the electron cluster and is corrected for leakage from the electron cluster itself and for energy deposits from pile-up. The tracking isolation variable is based on the track $p_{\mathrm {T}}$ sum around the electron in a cone of size $\Delta R < 0.3$, excluding the electron track. In every $p_{\mathrm {T}}$ bin both requirements are chosen to result separately in a 90 % electron selection efficiency for prompt electrons from *Z* boson decays.

Muon candidates are defined by matching tracks in the muon spectrometer with tracks in the inner detector. The track $p_{\mathrm {T}}$ is determined through a global fit of the hits which takes into account the energy loss in the calorimeters. The track is required to have $|z_0|<2$ mm and a transverse impact parameter significance, $|d_0/\sigma (d_0)|<3$, consistent with originating in the hard interaction. Muons are required to have $p_{\mathrm {T}}>25\,\mathrm {{\mathrm {GeV}}{}}$ and be within $|\eta |<2.5$. To reduce the background from muons originating from heavy-flavour decays inside jets, muons are required to be separated by $\Delta R>0.4$ from the nearest jet, and to be isolated. They are required to satisfy the isolation requirement $I^{\ell }< 0.05$, where the isolation variable is the ratio of the sum of $p_{\mathrm {T}}$ of tracks, excluding the muon, in a cone of variable size $\Delta R = 10\,\mathrm {{\mathrm {GeV}}{}}/p_{\mathrm {T}}(\mu )$ to the $p_{\mathrm {T}}$ of the muon [46]. The isolation requirement has an efficiency of about 97 % for prompt muons from *Z* boson decays.

Jets are reconstructed using the anti-$k_{t}$ algorithm [47] implemented in the FastJet package [48] with radius parameter $R = 0.4$. The jet reconstruction starts from topological clusters calibrated and corrected for pile-up effects using the jet area method [49]. A residual correction dependent on the instantaneous luminosity and the number of reconstructed primary vertices in the event [50] is then applied. They are calibrated using an energy- and $\eta$-dependent simulation-based calibration scheme, with in situ corrections based on data [51] and are accepted if $p_{\mathrm {T}}> 25\,\mathrm {{\mathrm {GeV}}{}}$ and $|\eta | < 2.5$. To reduce the contribution from jets associated with pile-up, jets with $p_{\mathrm {T}}< 50\,\mathrm {{\mathrm {GeV}}{}}$ are required to satisfy $|\mathrm {JVF}| > 0.5$, where JVF is the ratio of the sum of the $p_{\mathrm {T}}$ of tracks associated with both the jet and the primary vertex, to the sum of $p_{\mathrm {T}}$ of all tracks associated with the jet. Jets with no associated tracks or with $|\eta | > 2.4$ at the edge of the tracker acceptance are always accepted.

To prevent double-counting of electron energy deposits as jets, the closest jet lying within $\Delta R < 0.2$ from a reconstructed electron is removed. To remove leptons from heavy-flavour decays, the lepton is discarded if the lepton is found to lie within $\Delta R < 0.4$ from a selected jet axis.

The purity of the selected $t\bar{t}$ sample is improved by tagging jets containing *b*-hadrons, exploiting their long decay time and the large mass. Information from the track impact parameters, secondary vertex location and decay topology are combined in a neural-network-based algorithm (MV1) [52]. The operating point used corresponds to an overall 70 % *b*-tagging efficiency in $t\bar{t}$ events, and to a probability to mis-identify light-flavour jets of approximately 1 %.

The missing transverse momentum $E_{\mathrm {T}}^\mathrm{miss}$ is computed from the vector sum of the transverse momenta of the reconstructed calibrated physics objects (electrons, photons, hadronically decaying $\tau$ leptons, jets and muons) as well as the transverse energy deposited in the calorimeter cells not associated with these objects [53]. Calorimeter cells not associated with any physics object are calibrated using tracking information before being included in the $E_{\mathrm {T}}^\mathrm{miss}$ calculation. The contribution from muons is added using their momentum. To avoid double counting of energy, the parameterized muon energy loss in the calorimeters is subtracted in the $E_{\mathrm {T}}^\mathrm{miss}$ calculation.

**Fig. 1:**
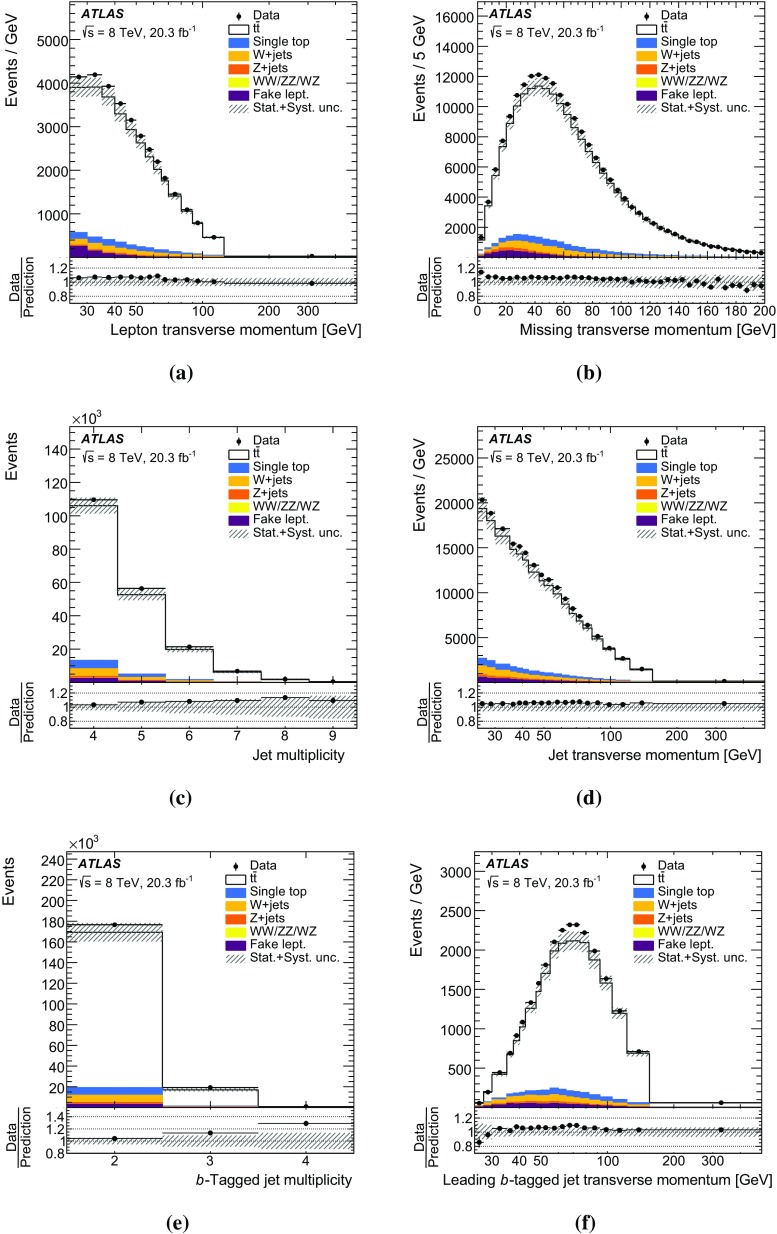
Kinematic distributions of the combined electron and muon selections at the detector level: **a** lepton transverse momentum and **b** missing transverse momentum $E_{\mathrm {T}}^\mathrm{miss}$, **c** jet multiplicity, **d** jet transverse momentum, **e** *b*-tagged jet multiplicity and **f** leading *b*-tagged jet $p_{\mathrm {T}}$. Data distributions are compared to predictions using Powheg +Pythia as the $t\bar{t}$ signal model. The *hashed area* indicates the combined statistical and systematic uncertainties on the total prediction, excluding systematic uncertainties related to the modelling of the $t\bar{t}$ system

### Event selection at detector level

The event selection consists of a set of requirements based on the general event quality and on the reconstructed objects, defined above, that characterize the final-state event topology. Each event must have a reconstructed primary vertex with five or more associated tracks. The events are required to contain exactly one reconstructed lepton candidate with $p_{\mathrm {T}}> 25\,\mathrm {{\mathrm {GeV}}{}}$ geometrically matched to a corresponding object at the trigger level and at least four jets with $p_{\mathrm {T}}> 25\,\mathrm {{\mathrm {GeV}}{}}$ and $|\eta | < 2.5$. At least two of the jets have to be tagged as *b*-jets. The event selection is summarized in Table 1. The event yields are displayed in Table 2 for data, simulated signal, and backgrounds (the background determination is described in Sect. 6). Figure 1 shows, for some key distributions, the comparison between data and predictions normalized to the data integrated luminosity. The selection produces a quite clean $t\bar{t}$sample, the total background being at the 10 % level. The difference between data and predicted event yield is $\sim$7 %, in fair agreement with the theoretical uncertainty on the $t\bar{t}$total cross-section used to normalize the signal MC simulation (see Sect. 3).

**Table 1:** Summary of all requirements included in the event selection

Cut	Event selection
Single lepton trigger	Electrons (isolated): $p_{\mathrm {T}}$ > 60 (24) ${\mathrm {GeV}}$
	Muons (isolated): $p_{\mathrm {T}}$ > 36 (24) ${\mathrm {GeV}}$
Primary vertex	$\ge$5 tracks with $p_{\mathrm {T}}$ $>0.4\,$ ${\mathrm {GeV}}$
Exactly one isolated lepton	Muons: $p_{\mathrm {T}}$ $> 25\,$ ${\mathrm {GeV}}$, $|\eta | < 2.5$
	Electrons: $p_{\mathrm {T}}$ > $25\,$ ${\mathrm {GeV}}$
	$|\eta |< 2.47$, excluding $1.37< |\eta | < 1.52$
Jets	$\ge$4 jets $p_{\mathrm {T}}$ $> 25\,$ ${\mathrm {GeV}}$, $|\eta | < 2.5$
	$\ge$2 *b*-tagged jets at $\epsilon _b=70$ %

**Table 2:** Event yields in the $e+$jets and $\mu +$jets channels after the selection. The signal model, denoted $t\bar{t}$ in the table, is generated using Powheg +Pythia . The quoted uncertainties represent the sum in quadrature of the statistical and systematic uncertainties on each subsample. Neither modelling uncertainties nor uncertainties on the inclusive ${t\bar{t}}$ cross-section are included in the systematic uncertainties

	$e+$jets	$\mu +$jets
$t\bar{t}$	74,000 ± 4700	92,000 ± 5900
Single top	3600 ± 200	4400 ± 300
$W+$jets	3000 ± 300	4400 ± 400
*Z*+jets	1100 ± 600	570 ± 300
*WW*/*WZ*/*ZZ*	73 ± 40	67 ± 35
Non-prompt and fake lept.	2000 ± 900	1400 ± 600
Prediction	84,000 ± 4900	103,000 ± 6000
Data	89,413	108,131

### Particle-level objects and fiducial phase-space definition

Particle-level objects are defined for simulated events in analogy to the detector-level objects described above. Only stable final-state particles, i.e. particles that are not decayed further by the generator, and unstable particles^2^ that are to be decayed later by the detector simulation, are considered.

The fiducial phase space for the measurements presented in this paper is defined using a series of requirements applied to particle-level objects close to those used in the selection of the detector-level objects. The procedure explained in this section is applied to the $t\bar{t}$ signal only, since the background subtraction is performed before unfolding the data.

Electrons and muons must not originate, either directly or through a $\tau$ decay, from a hadron in the MC particle record. This ensures that the lepton is from an electroweak decay without requiring a direct match to a *W* boson. The four-momenta of leptons are modified by adding the four-momenta of all photons within $\Delta R=0.1$ that do not originate from hadron decays to take into account final-state QED radiation. Such leptons are then required to have $p_{\mathrm {T}}> 25\,\mathrm {{\mathrm {GeV}}{}}$ and $|\eta | < 2.5$. Electrons in the transition region ($1.37< \eta < 1.52$ ) are rejected at the detector level but accepted in the fiducial selection. This difference is accounted for by the efficiency correction described in Sect. 8.1.

The particle-level missing transverse momentum is calculated from the four-vector sum of the neutrinos, discarding neutrinos from hadron decays, either directly or through a $\tau$ decay. Particle-level jets are clustered using the anti-$k_{t}$ algorithm with radius parameter $R = 0.4$, starting from all stable particles, except for selected leptons (*e*, $\mu$, $\nu$) and the photons radiated from the leptons. Particle-level jets are required to have $p_{\mathrm {T}}> 25\,\mathrm {{\mathrm {GeV}}{}}$ and $|\eta | < 2.5$. Hadrons containing a *b*-quark with $p_{\mathrm {T}}> 5\,\mathrm {{\mathrm {GeV}}{}}$ are associated with jets through a ghost matching technique as described in Ref. [49]. Particle *b*-tagged jets have $p_{\mathrm {T}}> 25\,\mathrm {{\mathrm {GeV}}{}}$ and $|\eta | < 2.5$. The events are required to contain exactly one reconstructed lepton candidate with $p_{\mathrm {T}}> 25\,\mathrm {{\mathrm {GeV}}{}}$ and at least four jets with $p_{\mathrm {T}}> 25\,\mathrm {{\mathrm {GeV}}{}}$ and $|\eta | < 2.5$. At least two of the jets have to be *b*-tagged. Dilepton events where only one lepton passes the fiducial selection are by definition included in the fiducial measurement.

### Parton-level objects and full phase-space definition

Parton-level objects are defined for simulated events. Only top quarks decaying directly to a *W* boson and a *b*-quark in the simulation are considered.^3^ The full phase space for the measurements presented in this paper is defined by the set of ${t\bar{t}}$ pairs in which one top quark decays semileptonically (including $\tau$ leptons) and the other decays hadronically. Events in which both top quarks decay semileptonically define the dilepton background, and are thus removed from the signal simulation.

## Kinematic reconstruction

The pseudo-top algorithm [4] reconstructs the kinematics of the top quarks and their complete decay chain from final-state objects, namely the charged lepton (electron or muon), missing transverse momentum, and four jets, two of which are *b*-tagged. By running the same algorithm on detector- and particle-level objects, the degree of dependency on the details of the simulation is strongly reduced compared to correcting to parton-level top quarks.

In the following, when more convenient, the leptonically (hadronically) decaying *W* boson is referred to as the leptonic (hadronic) *W* boson, and the semileptonically (hadronically) decaying top quark is referred to as the leptonic (hadronic) top quark.

The algorithm starts with the reconstruction of the neutrino four-momentum. The *z*-component of the neutrino momentum is calculated using the *W* boson mass constraint imposed on the invariant mass of the system of the charged lepton and the neutrino. If the resulting quadratic equation has two real solutions, the one with smallest absolute value of $|p_z|$ is chosen. If the determinant is negative, only the real part is considered. The leptonic *W* boson is reconstructed from the charged lepton and the neutrino and the leptonic top quark is reconstructed from the leptonic *W* and the $b\text{-tagged }$ jet closest in $\Delta R$ to the charged lepton. The hadronic *W* boson is reconstructed from the two non-*b*-tagged jets whose invariant mass is closest to the mass of the *W* boson. This choice yields the best performance of the algorithm in terms of the correlation between detector, particle and parton levels. Finally, the hadronic top quark is reconstructed from the hadronic *W* boson and the other $b\text{-jet }$. In events with more than two *b*-tagged jets, only the two with the highest transverse momentum are considered.

## Background determination

The single-top-quark background is the largest background contribution, amounting to approximately 4 % of the total event yield and 40 % of the total background estimate.

The shape of the distributions of the kinematical variables of this background is evaluated with a Monte Carlo simulation, and the event yields are normalized to the most recent calculations of their cross-sections, as described in Sect. 3. The overlap between the *Wt* and ${t\bar{t}}$ samples is handled using the diagram removal scheme [54].

The *W*+jets background represents the second largest background. After the event selection, approximately 3–4 % of the total event yield and 35 % of the total background estimate is due to *W*+jets events. The estimation of this background is performed using a combination of MC simulation and data-driven techniques. The Alpgen+Pythia *W*+jets samples, normalized to the inclusive *W* boson NNLO cross-section, are used as a starting point while the absolute normalization and the heavy-flavour fractions of this process, which are affected by large theoretical uncertainties, are determined from data.

The corrections for generator mis-modelling in the fractions of *W* boson production associated with jets of different flavour components ($W+b\bar{b}$, $W+c\bar{c}$, $W+c$) are estimated in a sample with the same lepton and $E_{\mathrm {T}}^{\mathrm {miss}}$ selections as the signal selection, but with only two jets and no *b*-tagging requirements. The *b*-jet multiplicity, in conjunction with knowledge of the *b*-tagging and mis-tag efficiency, is used to extract the heavy-flavour fraction. This information is extrapolated to the signal region using MC simulation, assuming constant relative rates for the signal and control regions.

The overall *W*+jets normalization is then obtained by exploiting the expected charge asymmetry in the production of $W^+$ and $W^-$ bosons in *pp* collisions. This asymmetry is predicted by theory [55] and evaluated using MC simulation, while other processes in the $t\bar{t}$ sample are symmetric in charge except for a small contamination from single-top and *WZ* events, which is subtracted using MC simulation. The total number of *W*+jets events in the sample can thus be estimated with the following equation:

1 $$\begin{aligned} N_{W^+} + N_{W^-} = \left( \frac{r_\mathrm{MC} + 1}{r_\mathrm{MC} - 1}\right) (D_\mathrm{+} - D_\mathrm{-}), \end{aligned}$$

where $r_\mathrm{MC}$ is the ratio of the number of events with positive leptons to the number of events with negative leptons in the MC simulation, and $D_\mathrm{+}$ and $D_\mathrm{-}$ are the number of events with positive and negative leptons in the data, respectively.

Multi-jet production processes have a large cross-section and mimic the lepton+jets signature due to jets misidentified as prompt leptons (fake leptons) or semileptonic decays of heavy-flavour hadrons (non-prompt real leptons). This background is estimated directly from data by using the matrix-method technique [56]. The number of background events in the signal region is evaluated by applying efficiency factors to the number of events passing the tight (signal) and loose selection. The fake leptons efficiency is measured using data in control regions dominated by the multi-jet background with the real-lepton contribution subtracted using MC simulation. The real leptons efficiency is extracted from a tag-and-probe technique using leptons from *Z* boson decays. Fake leptons events contribute to the total event yield at approximately the 1–2 % level.

*Z*+jets and diboson events are simulated with MC generators, and the event yields are normalized to the most recent theoretical calculation of their cross-sections. The total contribution of these processes is less than 1 % of the total event yield or approximatively 10 % of the total background.

Top-quark pair events with both top quarks and anti-top quarks decaying semileptonically (including decays to $\tau$) can sometimes pass the event selection, contributing approximately 5 % to the total event yield. The fraction of dileptonic $t\bar{t}$ events in each $p_{\mathrm {T}}$ bin is estimated with the same MC sample used for the signal modelling. In the fiducial phase-space definition, semileptonic top-quark decays to $\tau$ leptons in lepton+jets $t\bar{t}$ events are considered as signal only if the $\tau$ lepton decays leptonically.

## Observables

A set of measurements of the $t\bar{t}$  production cross-sections is presented as a function of kinematic observables. In the following, the indices *had* and *lep* refer to the hadronically and semileptonically decaying top quarks, respectively. The indices 1 and 2 refer respectively to the leading and sub-leading top quark, ordered by transverse momentum.

**Fig. 2:**
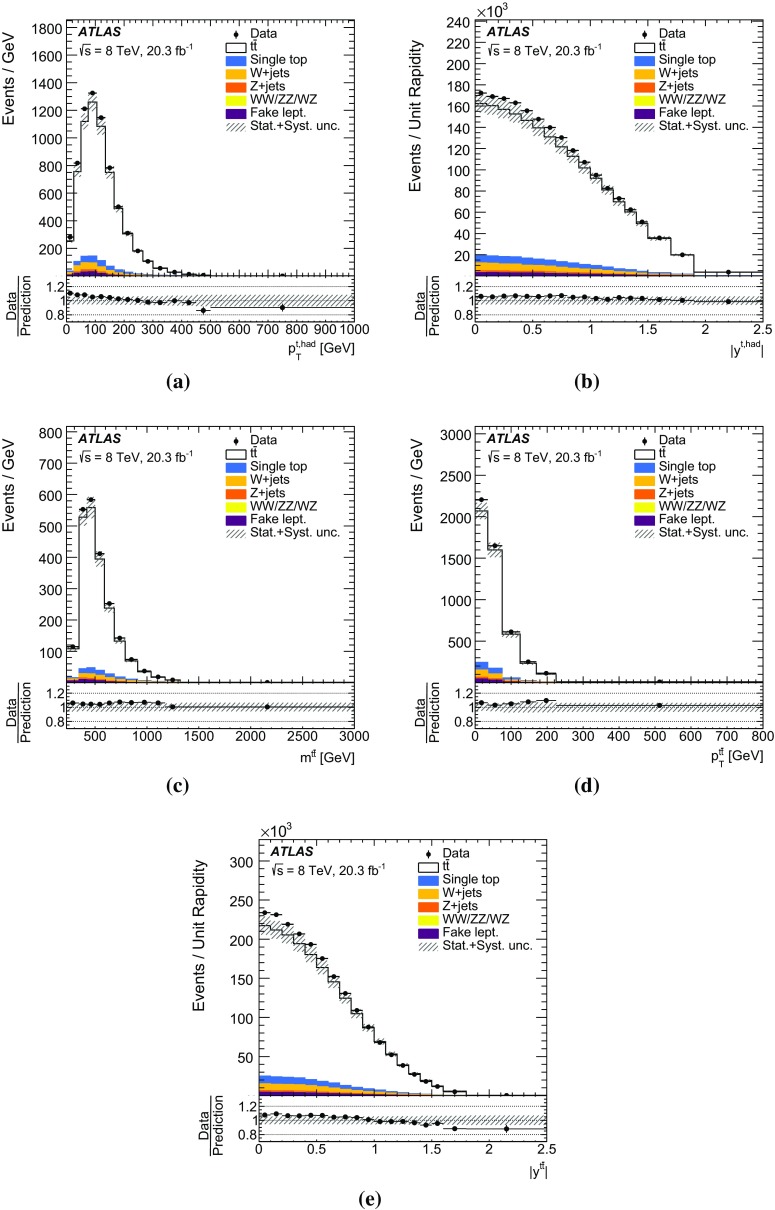
Distributions of observables of the combined electron and muon selections at detector level: **a** hadronic top-quark transverse momentum $p_{\mathrm {T}}^{t,\mathrm{had}}$ and **b** absolute value of the rapidity $|y^{t,\mathrm{had}}|$ , **c** ${t\bar{t}}$  invariant mass $m^{t\bar{t}}$, **d** transverse momentum $p_{\mathrm {T}}^{t\bar{t}}$ and **e** absolute value of the rapidity $|y^{t\bar{t}}|$ . Data distributions are compared to predictions, using Powheg +Pythia as the $t\bar{t}$ signal model. The *hashed area* indicates the combined statistical and systematic uncertainties (described in Sect. 9) on the total prediction, excluding systematic uncertainties related to the modelling of the $t\bar{t}$ system

First, a set of baseline observables is presented: transverse momentum ($p_{\mathrm {T}}^{t,\mathrm{had}}$) and absolute value of the rapidity ($|y^{t,\mathrm{had}}|$) of the hadronically decaying top quark (which was chosen over the leptonic top quark due to better resolution), and the transverse momentum ($p_{\mathrm {T}}^{t\bar{t}}$), absolute value of the rapidity ($|y^{t\bar{t}}|$) and invariant mass ($m^{t\bar{t}}$) of the $t\bar{t}$ system. These observables, shown in Fig. 2, have been previously measured by the ATLAS experiment using the 7  ${\mathrm {TeV}}$ dataset [3, 4] except for $|y^{t,\mathrm{had}}|$ which has not been measured in the full phase-space. The level of agreement between data and prediction is within the quoted uncertainties for $|y^{t,\mathrm{had}}|$, $m^{t\bar{t}}$ and $p_{\mathrm {T}}^{t\bar{t}}$. A trend is observed in the $p_{\mathrm {T}}^{t,\mathrm{had}}$ distribution, which is not well modelled at high values. A fair agreement between data and simulation is observed for large absolute values of the ${t\bar{t}}$ rapidity.

Furthermore, angular variables sensitive to a $p_{\mathrm {T}}$ imbalance in the transverse plane, i.e. to the emission of radiation associated with the production of the top-quark pair, are employed to emphasize the central production region [8]. The angle between the two top quarks has been found to be sensitive to non-resonant contributions due to hypothetical new particles exchanged in the *t*-channel [7]. The rapidities of the two top quarks in the laboratory frame are denoted by $y^{t,\mathrm{1}}$ and $y^{t,\mathrm{2}}$, while their rapidities in the $t\bar{t}{}$ centre-of-mass frame are ${y^{\star }}= \frac{1}{2}\left( y^{t,\mathrm{1}}-y^{t,\mathrm{2}} \right)$ and $-{y^{\star }}$. The longitudinal motion of the $t\bar{t}$  system in the laboratory frame is described by the rapidity boost $y_\mathrm{boost}^{{t\bar{t}}}=\frac{1}{2} \left[ y^{t,\mathrm{1}} + y^{t,\mathrm{2}} \right]$ and $\chi ^{{t\bar{t}}}=e^{2|{y^{\star }}|}$, which is closely related to the production angle. In particular, many signals due to processes not included in the Standard Model are predicted to peak at low values of $\chi ^{{t\bar{t}}}$ [7]. Finally, observables depending on the transverse momentum of the decay products of the top quark have been found to be sensitive to higher-order corrections [10, 11].

The following additional observables are measured:

- The absolute value of the azimuthal angle between the two top quarks ($\Delta \phi ^{{t\bar{t}}}$);
- the absolute value of the out-of-plane momentum ($|p_\mathrm{out}^{{t\bar{t}}}|$), i.e. the projection of top-quark three-momentum onto the direction perpendicular to a plane defined by the other top quark and the beam axis (*z*) in the laboratory frame [8]:
  2 $$\begin{aligned} |p_\mathrm{out}^{{t\bar{t}}}|= \left| \vec {p}^{~t, \mathrm{had}} \cdot \frac{\vec {p}^{~t,\mathrm{lep}} \times \hat{z}}{|\vec {p}^{~t,\mathrm{lep}}\times \hat{z}|} \right| ; \end{aligned}$$
- the longitudinal boost of the ${t\bar{t}}$ system in the laboratory frame ($y_\mathrm{boost}^{{t\bar{t}}}$) [7];
- the production angle between the two top quarks ($\chi ^{{t\bar{t}}}$) [7];
- the scalar sum of the transverse momenta of the two top quarks ($H_\mathrm{T}^{{t\bar{t}}}$) [10, 11]
- and the ratio of the transverse momenta of the hadronic *W* boson and the top quark from which it originates ($R_{Wt}$) [10, 11]
  3 $$\begin{aligned} R_{Wt}= p_{\mathrm {T}}^{W,\mathrm{had}} / p_{\mathrm {T}}^{t,\mathrm{had}}. \end{aligned}$$

**Fig. 3:**
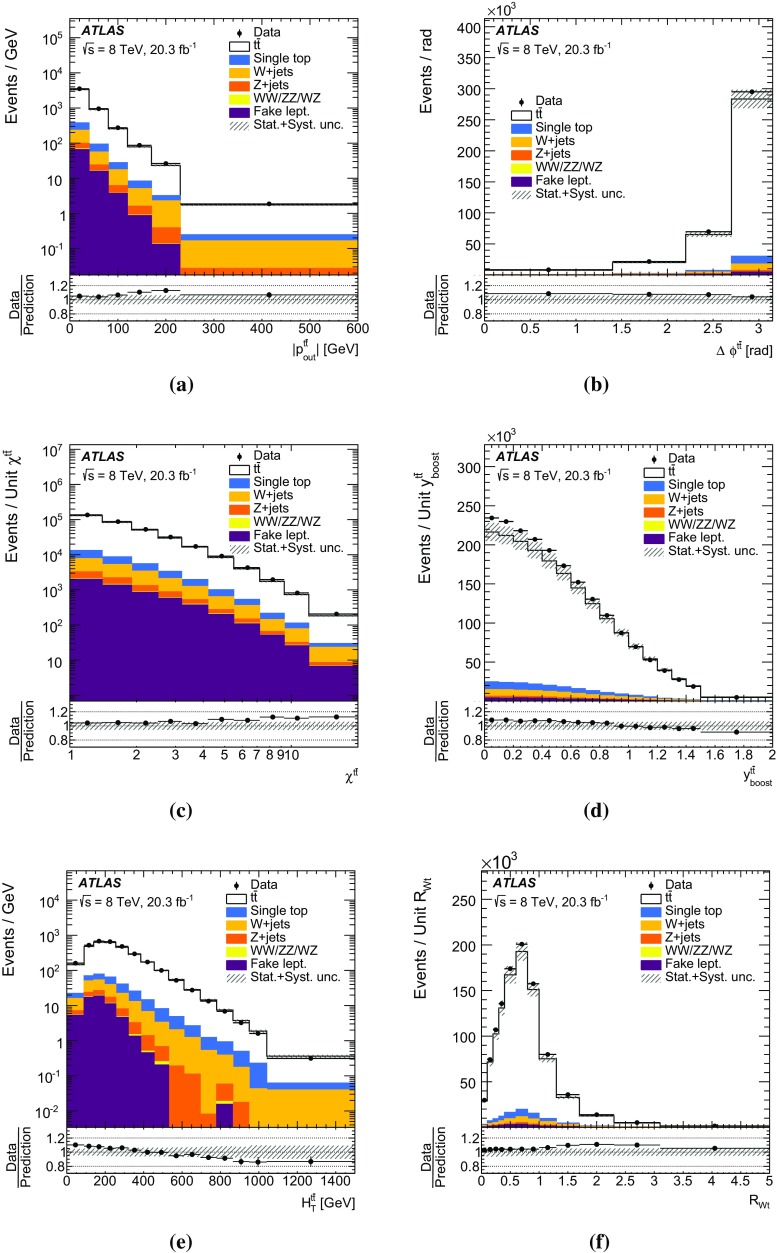
Distributions of observables of the combined electron and muon selections at the detector level: **a** absolute value of the out-of-plane momentum $p_\mathrm{out}^{{t\bar{t}}}$, **b** azimuthal angle between the two top quarks $\Delta \phi ^{{t\bar{t}}}$, **c** production angle $\chi ^{{t\bar{t}}}$, **d** longitudinal boost $y_\mathrm{boost}^{{t\bar{t}}}$, **e** scalar sum of hadronic and leptonic top-quarks transverse momenta and **f** ratio of the hadronic *W* boson and the hadronic top-quark transverse momenta. Data distributions are compared to predictions, using Powheg +Pythia as the $t\bar{t}$ signal model. The *hashed area* indicates the combined statistical and systematic uncertainties (described in Sect. 9) on the total prediction, excluding systematic uncertainties related to the modelling of the $t\bar{t}$ system

These observables are shown in Fig. 3 at detector level. All these variables show only modest agreement with data. In particular, at high values of $H_\mathrm{T}^{{t\bar{t}}}$, fewer events are observed with respect to the prediction. The longitudinal boost $y_\mathrm{boost}^{{t\bar{t}}}$ is predicted to be less central than the data. Finally, $R_{Wt}$ is predicted to be lower than observed in the range 1.5–3.0.

## Unfolding procedure

The underlying differential cross-section distributions are obtained from the detector-level events using an unfolding technique that corrects for detector effects. The iterative Bayesian method [57] as implemented in RooUnfold [58] is used. The individual $e+$jets and $\mu +$jets channels give consistent results and are therefore combined by summing the event yields before the unfolding procedure.

### Fiducial phase space

The unfolding starts from the detector-level event distribution ($N_\mathrm{reco}$), from which the backgrounds ($N_\mathrm{bg}$) are subtracted first. Next, the acceptance correction $f_\mathrm{acc}$ corrects for events that are generated outside the fiducial phase-space but pass the detector-level selection.

In order to separate resolution and combinatorial effects, distributions evaluated using a Monte Carlo simulation are corrected to the level where detector- and particle-level objects forming the pseudo-top quarks are angularly well matched. The matching correction $f_\mathrm{match}$ accounts for the corresponding efficiency. The matching is performed using geometrical criteria based on the distance $\Delta R$. Each particle *e* ($\mu$) is matched to the closest detector-level *e* ($\mu$) within $\Delta R < 0.02$. Particle-level jets are geometrically matched to the closest detector-level jet within $\Delta R < 0.4$. If a detector-level jet is not matched to a particle-level jet, it is assumed to be either from pile-up or matching inefficiency and is ignored. If two jets are reconstructed as being $\Delta R< 0.4$ from a single particle-level jet, the detector-level jet with smaller $\Delta R$ is matched to the particle-level jet and the other detector-level jet is unmatched.

**Fig. 4:**
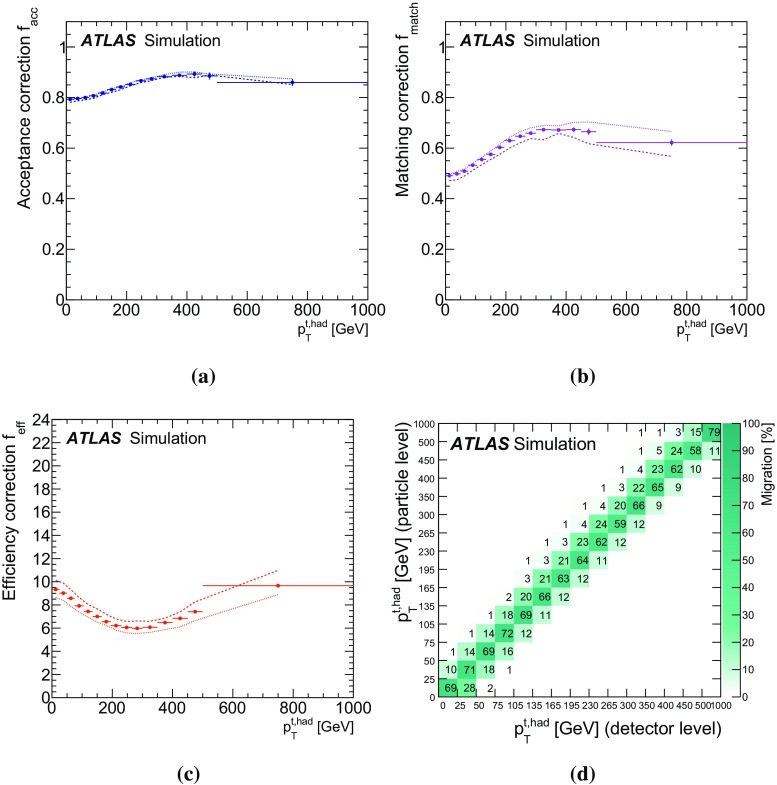
The **a** acceptance, **b** matching and **c** efficiency corrections, and the **d** detector-to-particle level migration matrix for the hadronic top-quark transverse momentum evaluated with the Powheg +Pythia simulation sample with $h_\mathrm{damp}\!=\!m_{t}$ and using CT10nlo PDF. In Fig. **a**–**c** the *dashed lines*illustrate the corrections evaluated on alternative ISR/FSR-varied samples. In Fig. **d**, the empty bins contain either no events or the number of events is less than 0.5 %

The unfolding step uses a migration matrix ($\mathcal {M}$) derived from simulated $t\bar{t}$ events which maps the binned generated particle-level events to the binned detector-level events. The probability for particle-level events to remain in the same bin is therefore represented by the elements on the diagonal, and the off-diagonal elements describe the fraction of particle-level events that migrate into other bins. Therefore, the elements of each row add up to unity as shown in Fig. 4d. The binning is chosen such that the fraction of events in the diagonal bins is always greater than 50 %. The unfolding is performed using four iterations to balance the goodness of fit and the statistical uncertainty. The effect of varying the number of iterations by one was tested and proved to be negligible. Finally, the efficiency correction $f_\mathrm{eff}$ corrects for events which pass the particle-level selection but are not reconstructed at the detector level.

All corrections are evaluated with simulation and are presented in Fig. 4 for the case of the $p_{\mathrm {T}}$ of the top quark decaying hadronically. This variable is particularly representative since the kinematics of the decay products of the top quark change substantially in the observed range. The decrease of the efficiency at high values is primarily due to the increasingly large fraction of non-isolated leptons and close or merged jets in events with high top-quark $p_{\mathrm {T}}$; in order to improve the selection efficiency in this boosted kinematic region, jets with larger *R* radius, with respect to the one used in this study, are required [59]. A similar effect is observed in the tail of the $t\bar{t}$ transverse momentum and rapidity, small $\Delta \phi ^{{t\bar{t}}}$ angle and high $H_\mathrm{T}^{{t\bar{t}}}$  distributions. The matching corrections reach the highest values, of the order of $f_\mathrm{match} = 0.6{-}0.7$, at low $t\bar{t}$ transverse momentum and large $t\bar{t}$ rapidity. Generally, the acceptance corrections are constant and close to unity, indicating very good correlation between the detector- and the particle-level reconstruction. This is also apparent from the high level of diagonality of the migration matrices, with correlations between particle and detector levels of 85–95 %.

The unfolding procedure for an observable *X* at particle level is summarized by the expression

4 $$\begin{aligned} \frac{\mathrm{d}\sigma ^\mathrm{fid}}{\mathrm{d}X^i} \equiv \frac{1}{\mathcal {L} \cdot \Delta X^i} \cdot f_\mathrm{eff}^i \cdot \sum _j \mathcal {M}_{ij}^{-1} \cdot f_\mathrm{match}^j \cdot f_\mathrm{acc}^j \cdot \left( N_\mathrm{reco}^j - N_\mathrm{bg}^j\right) \hbox {,} \end{aligned}$$

where the index *j* iterates over bins of *X* at detector level while the *i* index labels bins at particle level; $\Delta X^i$ is the bin width while $\mathcal {L}$ is the integrated luminosity and the Bayesian unfolding is symbolized by $\mathcal {M}_{ij}^{-1}$.

The integrated cross-section is obtained by integrating the unfolded cross-section over the kinematic bins, and its value is used to compute the normalized differential cross-section $1/\sigma ^\mathrm{fid}\cdot \mathrm{d}\sigma ^\mathrm{fid}/\mathrm{d}X^i$.

### Full phase space

The measurements are extrapolated to the full phase space of the ${t\bar{t}}$  system using a procedure similar to the one described in Sect. 8.1. The only difference is in the value used for the binning. The binning used by the CMS experiment in Ref. [5] is used for the observables measured by both experiments to facilitate future combinations. This binning is found to be compatible with the resolution of each observable. The fiducial phase-space binning is used for all the other observables. In order to unambiguously define leptonic and hadronic top quarks, the contribution of $t\bar{t}$ pairs decaying dileptonically is removed by applying a correction factor $\hat{f}_\mathrm{ljets}$ which represents the fraction of $t\bar{t}$ single-lepton events in the nominal sample. The $\tau$ leptons from the leptonically decaying *W* bosons are considered as signal regardless of the $\tau$ decay mode. The cross-section measurements are defined with respect to the top quarks before the decay (parton level) and after QCD radiation. Observables related to top quarks are extrapolated to the full phase-space starting from top quarks decaying hadronically at the detector level.

The acceptance correction $\hat{f}_\mathrm{acc}$ corrects for detector-level events which are reconstructed outside the parton-level bin range for a given variable. The migration matrix ($\hat{\mathcal {M}}$) is derived from simulated $t\bar{t}$ events decaying in the single-lepton channel and the efficiency correction $\hat{f}_\mathrm{eff}$ corrects for events which did not pass the detector-level selection.

The unfolding procedure is summarized by the expression

5 $$\begin{aligned} \frac{\mathrm{d}\sigma ^\mathrm{full}}{\mathrm{d}X^i} \equiv \frac{1}{\mathcal {L} \cdot \mathcal {B} \cdot \Delta X^i} \cdot \hat{f}_\mathrm{eff}^i \cdot \sum _j \hat{\mathcal {M}}_{ij}^{-1} \cdot \hat{f}_\mathrm{acc}^j \cdot \hat{f}_\mathrm{ljets}^i \cdot \left( N_\mathrm{reco}^j - N_\mathrm{bg}^j\right) \hbox {,} \end{aligned}$$

where the index *j* iterates over bins of observable *X* at the detector level while the *i* index labels bins at the parton level; $\Delta X^i$ is the bin width, $\mathcal {B}=0.438$ is the single-lepton branching ratio, $\mathcal {L}$ is the integrated luminosity and the Bayesian unfolding is symbolized by $\hat{\mathcal {M}}_{ij}^{-1}$.

The integrated cross-section is obtained by integrating the unfolded cross-section over the kinematic bins, and its value is used to compute the normalized differential cross-section $1/\sigma ^\mathrm{full}\cdot \mathrm{d}\sigma ^\mathrm{full} / \mathrm{d}X^i$.

To ensure that the results are not biased by the MC generator used for the unfolding procedure, a study is performed in which the particle- and parton-level spectra in simulation are altered by changing the shape of the distributions using continuous functions chosen depending on the observable. The studies confirm that these altered shapes are recovered within statistical uncertainties by the unfolding based on the nominal migration matrices.

## Uncertainties

This section describes the estimation of systematic uncertainties related to object reconstruction and calibration, MC generator modelling and background estimation.

To evaluate the impact of each uncertainty after the unfolding, the reconstructed distribution expected from simulation is varied. Corrections based on the nominal Powheg-Box signal sample are used to correct for detector effects and the unfolded distribution is compared to the known particle- or parton-level distribution. All detector- and background-related systematic uncertainties have been evaluated using the same generator, while alternative generators have been employed to assess modelling systematic uncertainties (e.g. different parton showers). In these cases the corrections, derived from the nominal generator, are used to unfold the detector-level spectra of the alternative generator. The relative difference between the unfolded spectra and the corresponding particle- or parton-level spectra of the alternative generator is taken as the uncertainty related to the generator modelling. After the unfolding, each distribution is normalized to unit area.

The covariance matrices for the normalized unfolded spectra due to the statistical and systematic uncertainties are obtained by evaluating the covariance between the kinematic bins using pseudo-experiments. In particular, the correlations due to statistical fluctuations for both data and the signal are evaluated by varying the event counts independently in every bin before unfolding, and then propagating the resulting variations through the unfolding.

### Object reconstruction and calibration

The jet energy scale uncertainty is derived using a combination of simulations, test beam data and in situ measurements [60–62]. Additional contributions from the jet flavour composition, calorimeter response to different jet flavours, and pile-up are taken into account. Uncertainties in the jet energy resolution are obtained with an in situ measurement of the jet response asymmetry in dijet events [63].

The efficiency to tag jets containing *b*-hadrons is corrected in simulation events by applying *b*-tagging scale factors, extracted in $t\bar{t}$ and dijet samples, in order to account for the residual difference between data and simulation. Scale factors are also applied for jets originating from light quarks that are mis-identified as *b*-jets. The associated systematic uncertainties are computed by varying the scale factors within their uncertainties [52, 64, 65].

The lepton reconstruction efficiency in simulation is corrected by scale factors derived from measurements of these efficiencies in data using a $Z \rightarrow \ell ^+ \ell ^-$ enriched control region. The lepton trigger and reconstruction efficiency scale factors, energy scale and resolution are varied within their uncertainties [66, 67].

The uncertainty associated with $E_{\mathrm {T}}^\mathrm{miss}$ is calculated by propagating the energy scale and resolution systematic uncertainties to all jets and leptons in the $E_{\mathrm {T}}^\mathrm{miss}$ calculation. Additional $E_{\mathrm {T}}^\mathrm{miss}$ uncertainties arising from energy deposits not associated with any reconstructed objects are also included [53].

### Signal modelling

The uncertainties of the signal modelling affect the kinematic properties of simulated $t\bar{t}$ events and reconstruction efficiencies.

To assess the uncertainty related to the generator, events simulated with MC@NLO +Herwig are unfolded using the migration matrix and correction factors derived from the Powheg +Herwig sample. The difference between the unfolded distribution and the known particle- or parton-level distribution of the MC@NLO +Herwig sample is assigned as the relative uncertainty for the fiducial or full phase-space distributions, respectively. This uncertainty is found to be in the range 2–5 %, depending on the variable, increasing up to 10 % at large $p_{\mathrm {T}}^{t}$, $m^{t\bar{t}}$, $p_{\mathrm {T}}^{t\bar{t}}$ and $|y^{t\bar{t}}|$. The observable that is most affected by these uncertainties is $m^{t\bar{t}}$ in the full phase space.

To assess the impact of different parton-shower models, unfolded results using events simulated with Powheg interfaced to Pythia are compared to events simulated with Powheg interfaced to Herwig, using the same procedure described above to evaluate the uncertainty related to the $t\bar{t}$generator. The resulting systematic uncertainties, taken as the symmetrized difference, are found to be typically at the 1–3 % level.

In order to evaluate the uncertainty related to the modelling of the ISR/FSR, $t\bar{t}$ MC samples with modified ISR/FSR modelling are used. The MC samples used for the evaluation of this uncertainty are generated using the Powheg generator interfaced to Pythia, where the parameters are varied as described in Sect. 3. This uncertainty is found to be in the range 2–5 %, depending on the variable of the $t\bar{t}$ system considered, and reaching the largest values at high $|y^{t}|$ and small $p_{\mathrm {T}}^{t\bar{t}}$.

The impact of the uncertainty related to the PDF is assessed by means of $t\bar{t}$ samples generated with MC@NLO interfaced to Herwig. An envelope of spectra is evaluated by reweighting the central prediction of the CT10nlo PDF set, using the full set of 52 eigenvectors at 68 % CL. This uncertainty is found to be less than 1 %.

As a check, the effect of the uncertainty on the top-quark mass was evaluated and found to affect only the efficiency correction by less than 1 %, consistent with what was observed by ATLAS for the analogous measurement with the 7  ${\mathrm {TeV}}$ data [4].

### Background modelling

Systematics affecting the background are modelled by adding to the signal spectrum the difference of the systematics-varied and nominal backgrounds.

The single-top background is assigned an uncertainty associated with the theoretical calculations used for its normalization [39–41]. The overall impact of this systematic uncertainty on the signal is around 0.5 %.

The systematic uncertainties due to the overall normalization and the heavy-flavour fraction of *W*+jets events are obtained by varying the data-driven scale factors within the statistical uncertainty of the *W*+jets MC sample. The *W*+jets shape uncertainty is extracted by varying the renormalization and matching scales in Alpgen. The *W*+jets MC statistical uncertainty is also taken into account. The overall impact of this uncertainty is less than 1 %.

The uncertainty on the background from non-prompt and fake-leptons is evaluated by varying the definition of loose leptons, changing the selection used to form the control region and propagating the statistical uncertainty of parameterizations of the efficiency to pass the tighter lepton requirements for real and fake leptons. The combination of all these components also affects the shape of the background. The overall impact of this systematic uncertainty is less than 1 %.

A 50 % uncertainty is applied to the normalization of the *Z*+jets background, including the uncertainty on the cross-section and a further 48 % due to uncertainties related to the requirement of the presence of at least four jets. A 40 % uncertainty is applied to the diboson background, including the uncertainty on the cross-section and a further 34 % due to the presence of two additional jets. The overall impact of these uncertainties is less than 1 %, and the largest contribution is due to the *Z*+jets background.

## Results

In this section, comparisons between unfolded data distributions and several SM predictions are presented for the different observables discussed in Sect. 7. Events are selected by requiring exactly one lepton and at least four jets with at least two of the jets tagged as originating from a *b*-quark. Normalized differential cross-sections are shown in order to remove systematic uncertainties on the normalization.

The SM predictions are obtained using different MC generators. The Powheg-Box generator [17], denoted “PWG” in the figures, is employed with three different sets of parton shower models, namely Pythia [19], Pythia8  [27] and Herwig [22]. The other NLO generator is MC@NLO [21] interfaced with the Herwig parton shower. Generators at the LO accuracy are represented by MadGraph  [29] interfaced with Pythia for parton showering, which calculates ${t\bar{t}}$ matrix elements with up to three additional partons and implements the matrix-element to parton-shower MLM matching scheme [30].

The level of agreement between the measured distributions and simulations with different theoretical predictions is quantified by calculating $\chi ^2$ values, employing the full covariance matrices, and inferring *p*-values (probabilities that the $\chi ^2$ is larger than or equal to the observed value) from the $\chi ^2$ and the number of degrees of freedom (NDF). Uncertainties on the predictions are not included. The normalization constraint used to derive the normalized differential cross-sections lowers by one unit the NDF and the rank of the $N_\mathrm{b} \times N_\mathrm{b}$ covariance matrix, where $N_\mathrm{b}$ is the number of bins of the spectrum under consideration [68]. In order to evaluate the $\chi ^2$ the following relation is used

6 $$\begin{aligned} \chi ^2 = V_{N_\mathrm{b}-1}^\mathrm{T} \cdot \mathrm{Cov}_{N_\mathrm{b}-1}^{-1} \cdot V_{N_\mathrm{b}-1}, \end{aligned}$$

where $V_{N_\mathrm{b}-1}$ is the vector of differences between data and prediction obtained by discarding one of the $N_\mathrm{b}$ elements and $\mathrm{Cov}_{N_\mathrm{b}-1}$ is the $(N_\mathrm{b}-1) \times (N_\mathrm{b}-1)$ sub-matrix derived from the full covariance matrix discarding the corresponding row and column. The sub-matrix obtained in this way is invertible and allows the $\chi ^2$ to be computed. The $\chi ^2$ value does not depend on the choice of the element discarded for the vector $V_{N_\mathrm{b}-1}$ and the corresponding sub-matrix $\mathrm{Cov}_{N_\mathrm{b}-1}$.

**Fig. 5:**
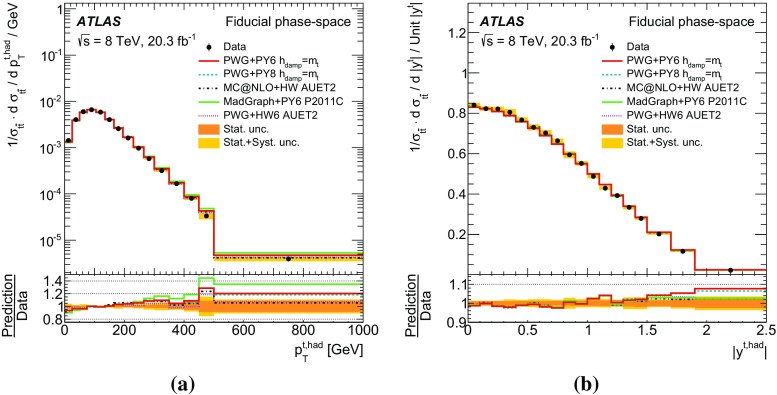
Fiducial phase-space normalized differential cross-sections as a function of the **a** transverse momentum ($p_{\mathrm {T}}^{t,\mathrm{had}}$) and **b** absolute value of the rapidity ($|y^{t,\mathrm{had}}|$) of the hadronic top quark. The *yellow* *bands* indicate the total uncertainty on the data in each bin. The Powheg +Pythia generator with $h_\mathrm{damp}\!=\!m_{t}$ and the CT10nlo PDF is used as the nominal prediction to correct for detector effects

**Fig. 6:**
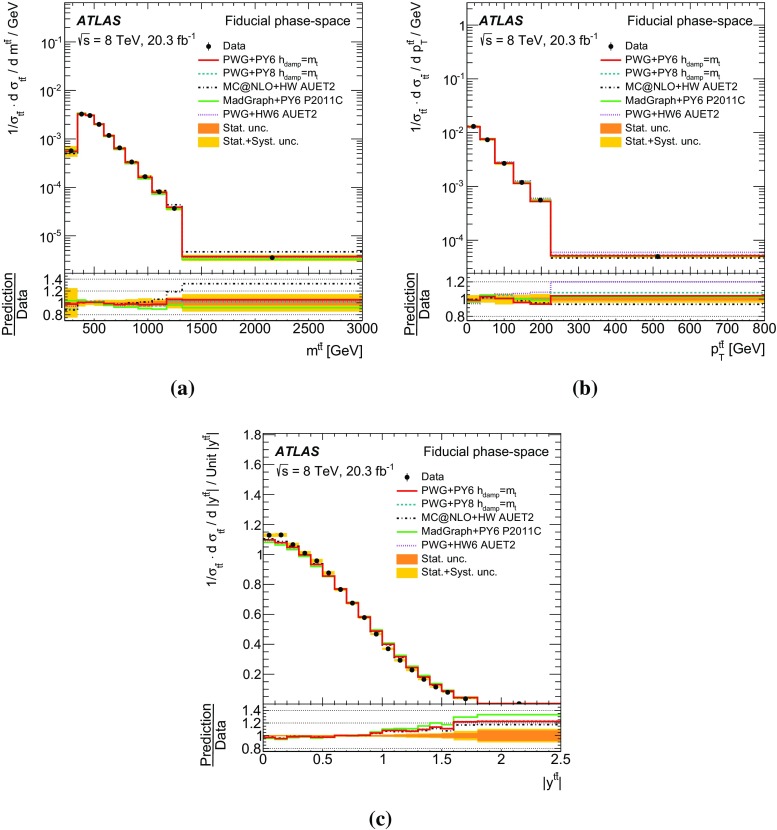
Fiducial phase-space normalized differential cross-sections as a function of the **a** invariant mass ($m^{t\bar{t}}$), **b** transverse momentum ($p_{\mathrm {T}}^{t\bar{t}}$) and **c** absolute value of the rapidity ($|y^{t\bar{t}}|$ ) of the ${t\bar{t}}$  system. The *yellow* *bands* indicate the total uncertainty on the data in each bin. The Powheg +Pythia generator with $h_\mathrm{damp}\!=\!m_{t}$ and the CT10nlo PDF is used as the nominal prediction to correct for detector effects

**Fig. 7:**
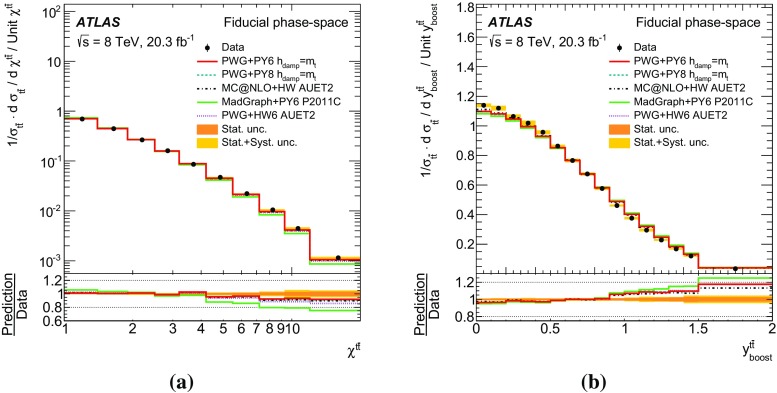
Fiducial phase-space normalized differential cross-sections as a function of the ${t\bar{t}}$  **a** production angle ($\chi ^{{t\bar{t}}}$) and **b** longitudinal boost ($y_\mathrm{boost}^{{t\bar{t}}}$). The *yellow bands* indicate the total uncertainty on the data in each bin. The Powheg +Pythia generator with $h_\mathrm{damp}\!=\!m_{t}$ and the CT10nlo PDF is used as the nominal prediction to correct for detector effects

**Fig. 8:**
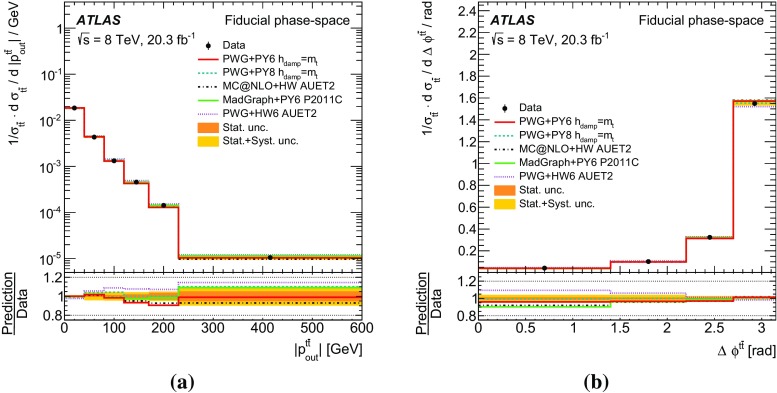
Fiducial phase-space normalized differential cross-sections as a function of the ${t\bar{t}}$  **a** out-of-plane momentum ($|p_\mathrm{out}^{{t\bar{t}}}|$) and **b** azimuthal angle ($\Delta \phi ^{{t\bar{t}}}$). The *yellow bands* indicate the total uncertainty on the data in each bin. The Powheg +Pythia generator with $h_\mathrm{damp}\!=\!m_{t}$ and the CT10nlo PDF is used as the nominal prediction to correct for detector effects

**Fig. 9:**
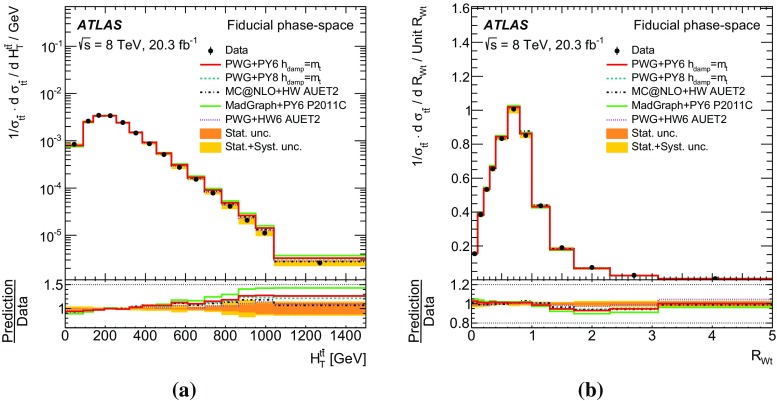
Fiducial phase-space normalized differential cross-sections as a function of the **a**  scalar sum of the transverse momenta of the hadronic and leptonic top quarks ($H_\mathrm{T}^{{t\bar{t}}}$) and **b**  the ratio of the hadronic *W* and the hadronic top transverse momenta ($R_{Wt}$). The *yellow bands* indicate the total uncertainty on the data in each bin. The Powheg +Pythia generator with $h_\mathrm{damp}\!=\!m_{t}$ and the CT10nlo PDF is used as the nominal prediction to correct for detector effects

The set of Figs. 5–9 presents the normalized $t\bar{t}$fiducial phase-space differential cross-sections as a function of the different observables. In particular, Fig. 5a, b show the distributions of the hadronic top-quark transverse momentum and the absolute value of the rapidity; Fig. 6a–c present the ${t\bar{t}}$ system invariant mass, transverse momentum, and absolute value of the rapidity, while the additional observables related to the $t\bar{t}$ system and the ratio of the transverse momenta of the hadronically decaying *W* boson and top quark are shown in Figs. 7, 8 and 9.

None of the predictions is able to correctly describe all the distributions, as also witnessed by the $\chi ^2$ values and the *p*-values listed in Table 3. In particular, a certain tension between data and all predictions is observed in the case of the hadronic top-quark transverse momentum distribution for values higher than about 400 ${\mathrm {GeV}}$. No electroweak corrections [69, 70, 70–73] are included in these predictions, as these have been shown to have a measurable impact only at very high values of the top quark transverse momentum, leading to a slightly softer $p_{\mathrm {T}}^{t,\mathrm{had}}$ spectrum as confirmed by the recent ATLAS measurement of the $t\bar{t}$differential distribution of the hadronic top-quark $p_{\mathrm {T}}$ for boosted top quarks [59]. The effect of electroweak corrections alone is not large enough to solve this discrepancy completely [59, 74]. The shape of the $|y^{t,\mathrm{had}}|$ distribution shows only a modest agreement for all the generators, with larger discrepancies observed in the forward region for Powheg +Pythia and Powheg +Pythia8.

For the $m^{t\bar{t}}$ distribution, the Powheg +Pythia, Powheg +Pythia8 and Powheg +Herwig generators are in better agreement with the data. All generators are in good agreement in the $p_{\mathrm {T}}^{t\bar{t}}$ spectrum except for Powheg +Herwig in the last bin. This observation suggests that setting $h_\mathrm{damp}\!=\!m_{t}$ in the Powheg samples improves the agreement at high values of the $t\bar{t}$  transverse momentum. The data at high values of ${t\bar{t}}$ rapidity is not adequately described by any of the generators considered. The same conclusions hold for the analogous distribution for the absolute spectra, although the overall agreement estimated with the $\chi ^2$ values and the *p*-values is better due to the larger uncertainties.

For the variables describing the hard-scattering interaction, the production angle $\chi ^{{t\bar{t}}}$ is well described in the central region. The forward region, described by the tail of this observable and by the tail of the longitudinal boost $y_\mathrm{boost}^{{t\bar{t}}}$, is not described correctly by any of the generators under consideration. For the variables describing the radiation along the $t\bar{t}$ pair momentum direction, both $|p_\mathrm{out}^{{t\bar{t}}}|$ and $\Delta \phi ^{{t\bar{t}}}$ indicate that the kinematics of top quarks produced in the collinear region ($\Delta \phi ^{{t\bar{t}}}$ $\lesssim \pi /2$) are described with fair agreement by all the generators, but the uncertainty is particularly large in this region. The tension observed in the $p_{\mathrm {T}}^{t,\mathrm{had}}$ spectrum is reflected in the tail of the $H_\mathrm{T}^{{t\bar{t}}}$ distribution. Finally, the ratio of the hadronic *W* boson and top-quark transverse momenta shows a mis-modelling in the range 1.5–3 for all the generators.

**Fig. 10:**
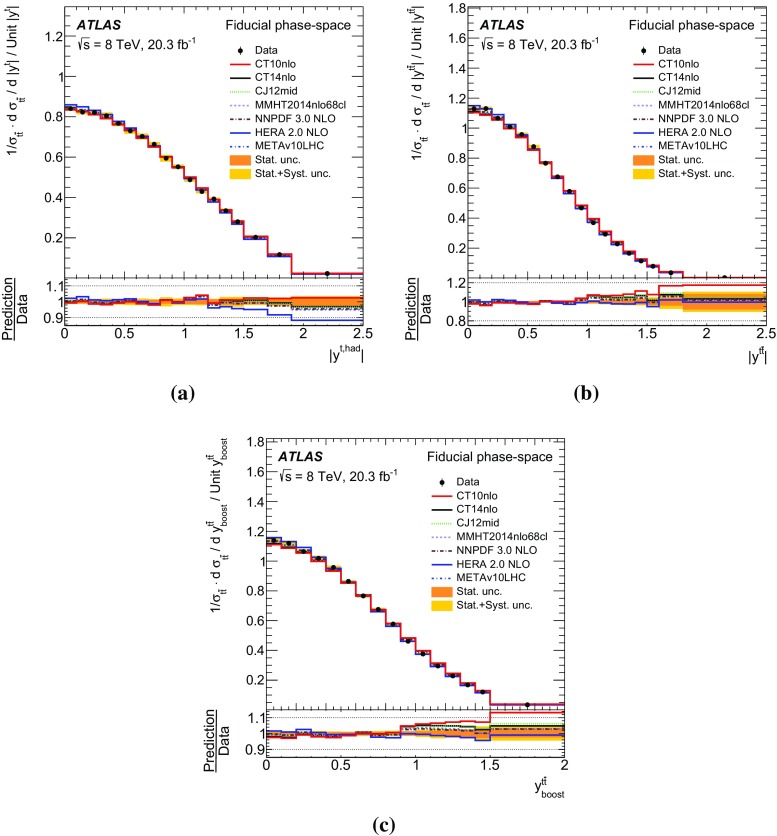
Fiducial phase-space normalized differential cross-sections as a function of the **a** absolute value of the rapidity of the hadronic top quark ($|y^{t,\mathrm{had}}|$), **b** absolute value of the rapidity ($|y^{t\bar{t}}|$ ) of the ${t\bar{t}}$  system and **c** longitudinal boost ($y_\mathrm{boost}^{{t\bar{t}}}$). The *yellow bands* indicate the total uncertainty on the data in each bin. The MC@NLO +Herwig generator is reweighted using the new PDF sets to produce the different predictions. The Powheg +Pythia generator with $h_\mathrm{damp}\!=\!m_{t}$ and the CT10nlo PDF is used as the nominal prediction to correct for detector effects

The difficulty in correctly predicting the data in the forward region was further investigated by studying the dependence of the predictions on different PDF sets. The study was performed for the rapidity observables $|y^{t,\mathrm{had}}|$ , $|y^{t\bar{t}}|$ and $y_\mathrm{boost}^{{t\bar{t}}}$, shown in Fig. 10 and comparing the data with the predictions of MC@NLO +Herwig for more recent sets of parton distribution functions. The results exhibit a general improvement in the description of the forward region for the most recent PDF sets (CT14nlo [75], CJ12mid [76], MMHT2014nlo [77], NNPDF 3.0 NLO [78], METAv10LHC [79], HERAPDF 2.0 NLO [80]). The improvement with respect to CT10nlo is also clearly shown in Table 5 which lists the $\chi ^2$ and corresponding *p*-values for the different sets. The only exception is represented by the $|y^{t,\mathrm{had}}|$  distribution using HERAPDF 2.0 NLO, for which a disagreement in the forward region is observed.

**Fig. 11:**
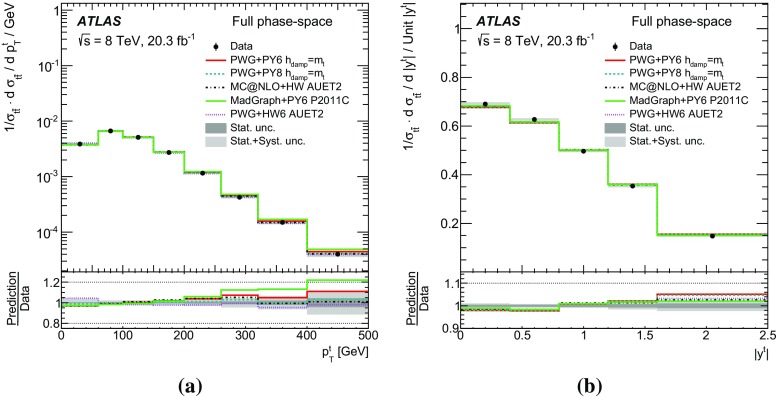
Full phase-space normalized differential cross-sections as a function of the **a** transverse momentum ($p_{\mathrm {T}}^{t}$) and **b** the absolute value of the rapidity ($|y^{t}|$ ) of the top quark. The *grey bands* indicate the total uncertainty on the data in each bin. The Powheg +Pythia generator with $h_\mathrm{damp}\!=\!m_{t}$ and the CT10nlo PDF is used as the nominal prediction to correct for detector effects

**Fig. 12:**
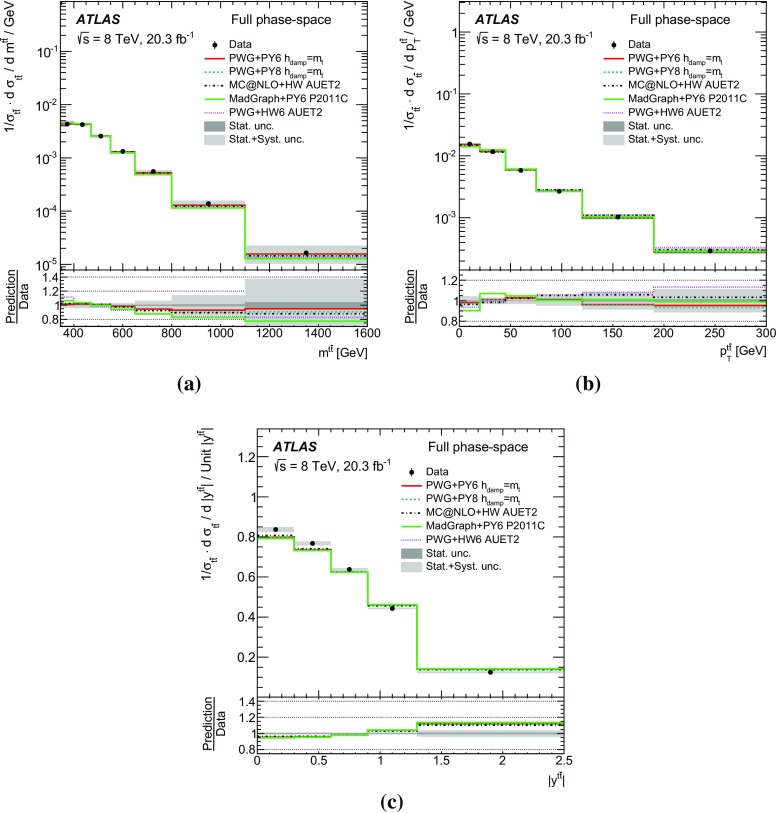
Full phase-space normalized differential cross-sections as a function of the  **a** invariant mass ($m^{t\bar{t}}$), **b** transverse momentum ($p_{\mathrm {T}}^{t\bar{t}}$) and **c** absolute value of the rapidity ($|y^{t\bar{t}}|$ ) of the ${t\bar{t}}$ system. The *grey bands* indicate the total uncertainty on the data in each bin. The Powheg +Pythia generator with $h_\mathrm{damp}\!=\!m_{t}$ and the CT10nlo PDF is used as the nominal prediction to correct for detector effects

**Fig. 13:**
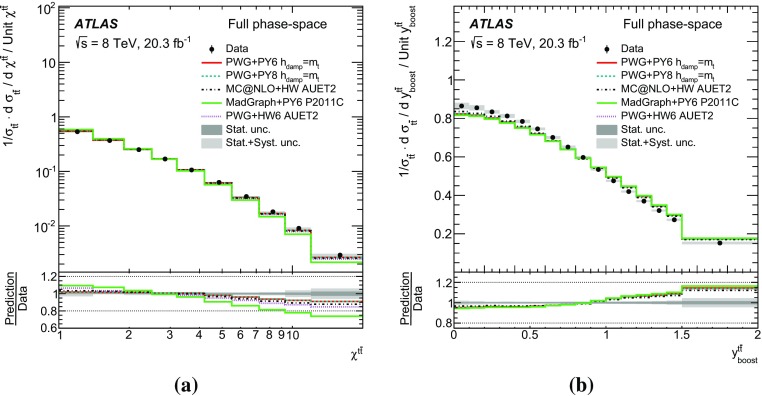
Full phase-space normalized differential cross-sections as a function of the  **a** production angle ($\chi ^{{t\bar{t}}}$) and **b** longitudinal boost ($y_\mathrm{boost}^{{t\bar{t}}}$) of the ${t\bar{t}}$ system. The *grey bands* indicate the total uncertainty on the data in each bin. The Powheg +Pythia generator with $h_\mathrm{damp}\!=\!m_{t}$ and the CT10nlo PDF is used as the nominal prediction to correct for detector effects

**Fig. 14:**
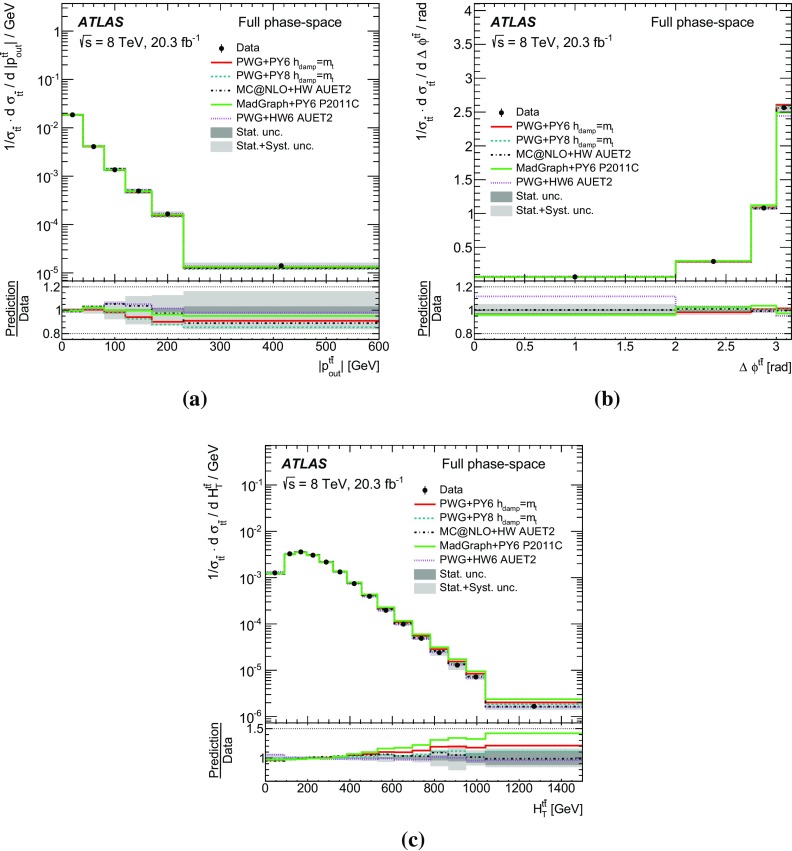
Full phase-space normalized differential cross-sections as a function of the  **a** out-of-plane momentum ($|p_\mathrm{out}^{{t\bar{t}}}|$), **b** azimuthal angle ($\Delta \phi ^{{t\bar{t}}}$), and **c**  scalar sum of the transverse momenta of the hadronic and leptonic top quarks ($H_\mathrm{T}^{{t\bar{t}}}$)) of the ${t\bar{t}}$ system. The *grey bands* indicate the total uncertainty on the data in each bin. The Powheg +Pythia generator with $h_\mathrm{damp}\!=\!m_{t}$ and the CT10nlo PDF is used as the nominal prediction to correct for detector effects

The set of Figs. 11–14 presents the normalized $t\bar{t}$ full phase-space differential cross-sections as a function of the different observables. In particular, Fig. 11a, b show the top-quark transverse momentum and the absolute value of the rapidity; Fig. 12a–c present the ${t\bar{t}}$ system invariant mass, transverse momentum and absolute value of the rapidity while the additional observables related to the $t\bar{t}$system are shown in Figs. 13 and 14. Regarding the comparison between data and predictions, the general picture, already outlined for the fiducial phase-space measurements, is still valid even though the uncertainties are much larger due to the full phase-space extrapolation. In particular, the predictions for the top-quark $p_{\mathrm {T}}$ and $H_\mathrm{T}^{{t\bar{t}}}$  tend to be in a better agreement with the data than what is observed in the fiducial phase-space. The $\chi ^2$ and corresponding *p*-values for the different observables and predictions are shown in Table 4.

**Fig. 15:**
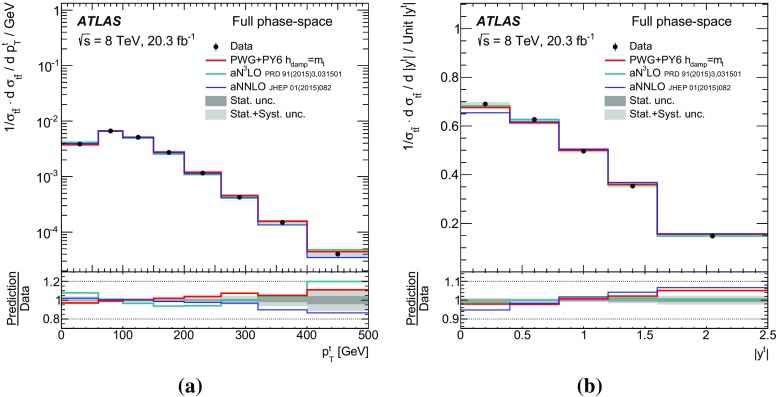
Full phase-space normalized differential cross-section as a function of the **a** transverse momentum ($p_{\mathrm {T}}^{t}$) and **b** absolute value of the rapidity of the top quark ($|y^{t}|$) compared to higher-order theoretical calculations. The *grey band* indicates the total uncertainty on the data in each bin. The Powheg +Pythia generator with $h_\mathrm{damp}\!=\!m_{t}$ and the CT10nlo PDF is used as the nominal prediction to correct for detector effects

**Fig. 16:**
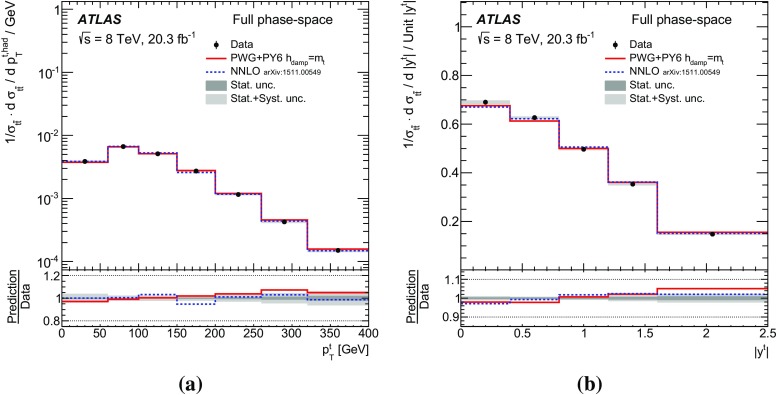
Full phase-space normalized differential cross-section as a function of the **a** transverse momentum ($p_{\mathrm {T}}^{t}$) and **b** absolute value of the rapidity of the top quark ($|y^{t}|$) compared to NNLO theoretical calculations [85] using the MSTW2008nnlo PDF set. The *grey band* indicates the total uncertainty on the data in each bin. The Powheg +Pythia generator with $h_\mathrm{damp}\!=\!m_{t}$ and the CT10nlo PDF is used as the nominal prediction to correct for detector effects

**Fig. 17:**
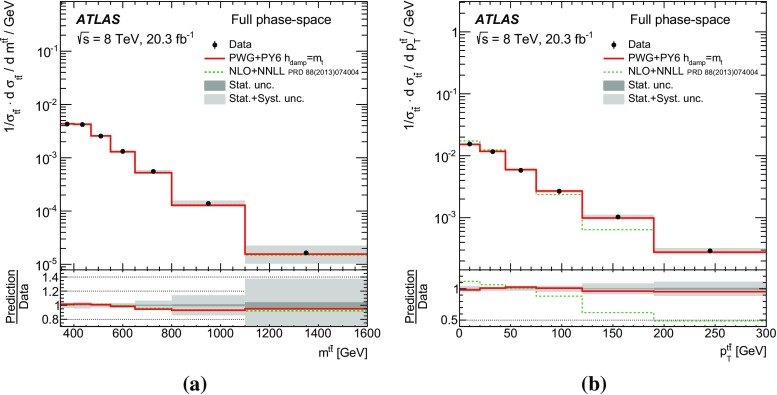
Full phase-space normalized differential cross-section as a function of the **a** invariant mass ($m^{t\bar{t}}$ ) and **b** transverse momentum ($p_{\mathrm {T}}^{t\bar{t}}$) of the $t\bar{t}$ system compared to higher-order theoretical calculations. The *grey band* indicates the total uncertainty on the data in each bin. The Powheg +Pythia generator with $h_\mathrm{damp}\!=\!m_{t}$ and the CT10nlo PDF is used as the nominal prediction to correct for detector effects

**Fig. 18:**
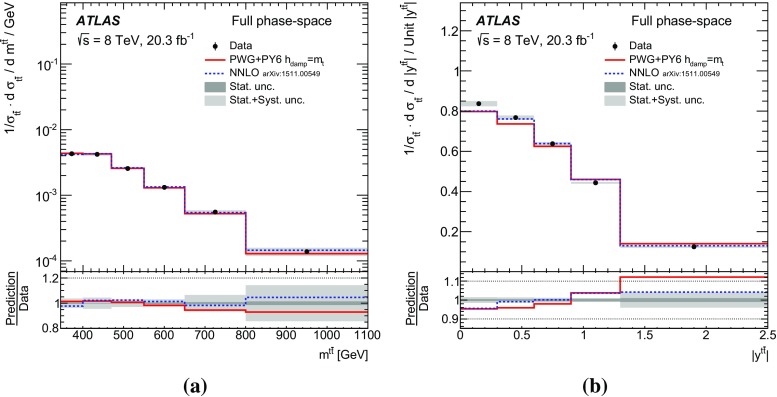
Full phase-space normalized differential cross-section as a function of the **a** invariant mass ($m^{t\bar{t}}$ ) and **b** absolute value of the rapidity ($|y^{t\bar{t}}|$) of the $t\bar{t}$ system compared to NNLO theoretical calculations [85] using the MSTW2008nnlo PDF set. The *grey band* indicates the total uncertainty on the data in each bin. The Powheg +Pythia generator with $h_\mathrm{damp}\!=\!m_{t}$ and the CT10nlo PDF is used as the nominal prediction to correct for detector effects

In Figs. 15–18 the normalized $t\bar{t}$full phase-space differential cross-section as a function of $p_{\mathrm {T}}^{t}$, $|y^{t}|$, $m^{t\bar{t}}$ and $|y^{t\bar{t}}|$ are compared with theoretical higher-order QCD calculations.

The measurements are compared to four calculations that offer beyond–NLO accuracy:

- an approximate next-to-next-to-leading-order (aNNLO) calculation based on QCD threshold expansions beyond the leading logarithmic approximation [81] using the CT14nnlo PDF [75];
- an approximate next-to-next-to-next-to-leading-order (aN $^3$ LO) calculation based on the resummation of soft-gluon contributions in the double-differential cross section at next-to-next-to-leading-logarithm (NNLL) accuracy in the moment-space approach in perturbative QCD [82] using the MSTW2008nnlo PDF [83];
- an approximate NLO+NNLL calculation [84] using the MSTW2008nnlo PDF [83].
- a full NNLO calculation [85] using the MSTW2008nnlo PDF [83]. The NNLO prediction does not cover the highest bins in $p_{\mathrm {T}}^{t}$ and $m^{t\bar{t}}$.

These predictions have been interpolated in order to match the binning of the presented measurements. Table 6 shows the $\chi ^2$ and *p*-values for these higher-order QCD calculations.

Figures 15 and 16 show a comparison of the $p_{\mathrm {T}}^{t}$ and $|y^{t}|$ distributions to the aNNLO and aN $^3$ LO, and to the NNLO calculations respectively. The aN $^3$ LO calculation is seen to improve the agreement compared to the Powheg +Pythia generator in $|y^{t}|$, but not in $p_{\mathrm {T}}^{t}$. The aNNLO prediction produces a $p_{\mathrm {T}}^{t}$ distribution that is softer than the data at high transverse momentum and does not improve the description of $|y^{t}|$. The NNLO calculation is in good agreement with both the $p_{\mathrm {T}}^{t}$ and $|y^{t}|$ distributions, in particular the disagreement seen at high $p_{\mathrm {T}}^{t}$ for the NLO generators is resolved by the NNLO calculation.

The measurement of the invariant mass and transverse momentum of the $t\bar{t}$ system is compared to the NLO+NNLL prediction in Fig. 17. The NLO+NNLL calculation shows a good agreement in the $m^{t\bar{t}}$ spectrum and a very large discrepancy for high values of the $t\bar{t}$ transverse momentum. Figure 18 shows a comparison of the NNLO calculation to the $m^{t\bar{t}}$ and $|y^{t\bar{t}}|$ measurements. For the rapidity of the $t\bar{t}$ system, the NNLO calculation improves the agreement slightly compared to the Powheg +Pythia prediction, but some shape difference can be seen between data and prediction.

**Table 3:** Comparison between the measured fiducial phase-space normalized differential cross-sections and the predictions from several MC generators. For each variable and prediction a $\chi ^2$ and a *p*-value are calculated using the covariance matrix of each measured spectrum. The number of degrees of freedom (NDF) is equal to $N_\mathrm{b}-1$ where $N_\mathrm{b}$ is the number of bins in the distribution

Variable	PWG+PY8		MC@NLO+HW		PWG+PY6		PWG+HW6		MadGraph+PY6	
	CT10 $h_\mathrm{damp}=m_{t}$		CT10 AUET2		CT10 $h_\mathrm{damp}=m_{t}$		CT10 $h_\mathrm{damp}=\infty$		P2011C	
	$\chi ^{2}$/NDF	*p*-value	$\chi ^{2}$/NDF	*p*-value	$\chi ^{2}$/NDF	*p*-value	$\chi ^{2}$/NDF	*p*-value	$\chi ^{2}$/NDF	*p*-value
$p_{T}^{t,\mathrm{had}}$	9.5/14	0.80	13/14	0.56	11/14	0.68	4.8/14	0.99	41/14	0.01
$R_{Wt}$	16/11	0.14	14/11	0.23	21/11	0.03	5.6/11	0.90	48/11	0.01
$\chi ^{t\bar{t}}$	18/9	0.04	24/9	$<0.01$	17/9	0.04	34/9	$<0.01$	130/9	0.01
$|y^{t\bar{t}}|$	35/17	0.01	25/17	0.10	31/17	0.02	33/17	0.01	58/17	0.01
$m^{t\bar{t}}$	17/10	0.08	33/10	0.01	11/10	0.38	16/10	0.11	18/10	0.05
$y_{boost}^{t\bar{t}}$	39/15	0.01	25/15	0.06	35/15	0.01	38/15	0.01	65/15	0.01
$|p_{out}^{t\bar{t}}|$	3.4/5	0.63	3.1/5	0.69	7.7/5	0.18	5.6/5	0.35	5.9 /5	0.31
$|y^{t,\mathrm{had}}|$	19/17	0.33	13/17	0.75	17/17	0.47	14/17	0.69	13/ 17	0.74
$p_{T}^{t\bar{t}}$	4.2/5	0.52	4.0/5	0.54	8.7/5	0.12	14/5	0.01	4.6/5	0.47
$H_{T}^{t\bar{t}}$	16/14	0.34	13/14	0.55	18/14	0.20	9.5/14	0.80	50/ 14	0.01
$\Delta \phi ^{t\bar{t}}$	0.3/3	0.96	3.7/3	0.29	1.2/3	0.74	5.4/3	0.14	6.0 /3	0.11

**Table 4:** Comparison between the measured full phase-space normalized differential cross-sections and the predictions from several MC generators. For each variable and prediction a $\chi ^2$ and a *p*-value are calculated using the covariance matrix of each measured spectrum. The number of degrees of freedom (NDF) is equal to $N_\mathrm{b}-1$ where $N_\mathrm{b}$ is the number of bins in the distribution

Variable	PWG+PY8		MC@NLO+HW		PWG+PY6		PWG+HW6		MadGraph+PY6	
	CT10 $h_\mathrm{damp}=m_{t}$		CT10 AUET2		CT10 $h_\mathrm{damp}=m_{t}$		CT10 $h_\mathrm{damp}=\infty$		MadGraph+PY6 P2011C	
	$\chi ^{2}$/NDF	*p*-value	$\chi ^{2}$/NDF	*p*-value	$\chi ^{2}$/NDF	*p*-value	$\chi ^{2}$/NDF	*p*-value	$\chi ^{2}$/NDF	*p*-value
$p_\mathrm{T}^{t}$	0.7/7	1.00	5.1/7	0.65	5.8/7	0.56	3.8/7	0.80	16/7	0.03
$\chi ^{t\bar{t}}$	29/9	0.01	69/9	$<0.01$	32/9	0.01	120/9	0.01	400/9	0.01
$|y^{t\bar{t}}|$	34/4	$<0.01$	24/4	0.01	35/4	0.01	33/4	0.01	44 /4	0.01
$m^{t\bar{t}}$	3.6/6	0.73	3.8/6	0.71	1.9/6	0.93	22/6	0.01	13/6	0.04
$y_\mathrm{boost}^{t\bar{t}}$	140/15	0.01	93/15	0.01	140/15	$<0.01$	140/15	$<0.01$	180/15	0.01
$|p_\mathrm{out}^{t\bar{t}}|$	1.8/5	0.88	1.9/5	0.86	1.1/5	0.96	2.5/5	0.78	0.8/5	0.98
$|y^{t}|$	2.3/4	0.69	1.5/4	0.83	2.5/4	0.65	1.8/4	0.77	1.2/4	0.87
$p_\mathrm{T}^{t\bar{t}}$	2.7/5	0.75	2.8/5	0.72	1.2/5	0.94	5.0/5	0.41	11/5	0.05
$H_\mathrm{T}^{t\bar{t}}$	3.2/14	1.00	7.3/14	0.92	16/14	0.29	3.2/14	1.00	44/14	0.01
$\Delta \phi ^{t\bar{t}}$	0.5/3	0.93	0.2/3	0.97	0.8/3	0.85	6.2/3	0.10	4.3/3	0.23

**Table 5:** Comparison between the measured fiducial phase-space normalized differential cross-sections and the predictions from new PDF sets using the MC@NLO +Herwig generator. For each variable and prediction a $\chi ^2$ and a *p*-value are calculated using the covariance matrix of each measured spectrum. The number of degrees of freedom (NDF) is equal to $N_\mathrm{b}-1$ where $N_\mathrm{b}$ is the number of bins in the distribution

Variable	CT14nlo		CJ12mid		MMHT2014nlo68cl		NNPDF30nlo		CT10nlo		METAv10LHC		HERA20NLO	
	$\chi ^{2}$/NDF	*p*-value	$\chi ^{2}$/NDF	*p*-value	$\chi ^{2}$/NDF	*p*-value	$\chi ^{2}$/NDF	*p*-value	$\chi ^{2}$/NDF	*p*-value	$\chi ^{2}$/NDF	*p*-value	$\chi ^{2}$/NDF	*p*-value
$|y^{t\bar{t}}|$	24/17	0.14	18/17	0.36	16/17	0.51	14/17	0.70	25/17	0.10	14/17	0.64	24/17	0.12
$|y^{t,\mathrm{had}}|$	15/17	0.60	13/17	0.71	14/17	0.66	12/17	0.79	13/17	0.75	13/17	0.71	26/17	0.08
$y_{boost}^{t\bar{t}}$	21/15	0.15	18/15	0.29	12/15	0.68	8.8/15	0.89	25/15	0.06	10/15	0.84	17/15	0.32

**Table 6:** Comparison between the measured full phase-space normalized differential cross-sections and higher-order QCD calculations. For each variable and prediction a $\chi ^2$ and a *p*-value are calculated using the covariance matrix of each measured spectrum. The number of degrees of freedom (NDF) is equal to $N_\mathrm{b}-1$ where $N_\mathrm{b}$ is the number of bins in the distribution

Variable	aN $^3$ LO		aNNLO	
	$\chi ^{2}$/NDF	*p*-value	$\chi ^{2}$/NDF	*p*-value
$p_{T}^{t}$	18/7	0.01	4.0/7	0.78
$|y^{t}|$	0.6/4	0.96	9.2/4	0.06

## Conclusions

Kinematic distributions of the top quarks in $t\bar{t}$ events, selected in the lepton+jets channel, are measured in the fiducial and full phase space using data from 8  ${\mathrm {TeV}}$ proton–proton collisions collected by the ATLAS detector at the Large Hadron Collider, corresponding to an integrated luminosity of 20.3 fb$^{-1}$. Normalized differential cross-sections are measured as a function of the hadronic top-quark transverse momentum and rapidity, and as a function of the mass, transverse momentum, and rapidity of the $t\bar{t}$ system. In addition, a new set of observables describing the hard-scattering interaction ($\chi ^{{t\bar{t}}}$, $y_\mathrm{boost}^{{t\bar{t}}}$) and sensitive to the emission of radiation along with the ${t\bar{t}}$ pair ($\Delta \phi ^{{t\bar{t}}}$, $|p_\mathrm{out}^{{t\bar{t}}}|$, $H_\mathrm{T}^{{t\bar{t}}}$, $R_{Wt}$) are presented.

The measurements presented here exhibit, for most distributions and in large part of the phase space, a precision of the order of 5 % or better and an overall agreement with the Monte Carlo predictions of the order of 10 %.

The $|y^{t\bar{t}}|$ and $y_\mathrm{boost}^{{t\bar{t}}}$distributions are not well modelled by any generator under consideration in the fiducial phase space, however the agreement improves when new parton distribution functions are used with the MC@NLO +Herwig generator.

All the generators under consideration consistently predict a ratio of the hadronic *W* boson and top-quark transverse momenta ($R_{Wt}$) with a mis-modelling of up to 10 % in the range 1.5–3.

The tail of the $p_{\mathrm {T}}^{t,\mathrm{had}}$ distribution is harder in all predictions than what is observed in data, an effect previously observed in measurements by ATLAS and CMS. The agreement improves when using the Herwig parton shower with respect to Pythia. The tension observed for Powheg +Pythia , Powheg +Pythia8   and MadGraph +Pythia in the $p_{\mathrm {T}}^{t}$ spectrum is reflected in the tail of the $H_\mathrm{T}^{{t\bar{t}}}$ distribution.

Similarly, both aN $^3$ LO and aNNLO predictions have a poor agreement in the $p_{\mathrm {T}}^{t}$ spectrum in the full phase space. However, the full NNLO calculation, which has just become available, is in good agreement with the $p_{\mathrm {T}}^{t}$ distribution, indicating the disagreement seen with the generators and other calculations is due to missing higher-order terms. The NNLO calculation also shows good agreement in the $|y^{t}|$ and $m^{t\bar{t}}$ distributions.

## Footnotes

1 ATLAS uses a right-handed coordinate system with its origin at the nominal interaction point (IP) in the centre of the detector and the *z*-axis along the beam pipe. The *x*-axis points from the IP to the centre of the LHC ring, and the *y*-axis points upward. Cylindrical coordinates (*r*,$\phi$) are used in the transverse plane, $\phi$ being the azimuthal angle around the beam pipe. The pseudorapidity is defined in terms of the polar angle $\theta$ as $\eta = - \ln \tan (\theta /2)$ and the angular separation between particles is defined as $\Delta R = \sqrt{(\Delta \phi )^2 + (\Delta \eta )^2}$.

2 Particles with a mean lifetime $\tau$ > 300 ps

3 These particles are labelled by a status code 155 in Herwig, 3 in Pythia and 22 in Pythia8 respectively.
